# SEDS glycosyltransferases in *E. faecalis* are upregulated by the CroS/R two-component system to enhance peptidoglycan synthesis during cell wall stress

**DOI:** 10.1128/msphere.00528-25

**Published:** 2025-11-24

**Authors:** Madison E. Nelson, Dušanka Djorić, Miryah E. Henriksen-Hadlock, Christopher J. Kristich

**Affiliations:** 1 Department of Microbiology and Immunology, Center for Infectious Disease Research, Medical College of Wisconsin 5506, Milwaukee, Wisconsin, USA; The University of Iowa, Iowa City, Iowa, USA

**Keywords:** SEDS glycosyltransferase, peptidoglycan synthesis, *Enterococcus faecalis*

## Abstract

Enterococci are commensals of the intestinal tract that are intrinsically resistant to cephalosporins, antibiotics that inhibit peptidoglycan synthesis. Prior treatment with cephalosporins is a risk factor for acquiring an enterococcal infection. We previously showed that FtsW, a SEDS (shape, elongation, division, and sporulation) protein, is essential for enterococcal cephalosporin resistance. SEDS proteins catalyze glycosyltransferase reactions to polymerize strands of peptidoglycan. Bacterial genomes typically only encode for two SEDS proteins, FtsW and RodA, that form the core of two different peptidoglycan synthases thought to function at distinct locations in the cell. However, a few bacterial genera, including enterococci, encode homologs of not only FtsW and RodA but also additional SEDS proteins. In general, very little is known about the function of these additional SEDS proteins. The genome of *Enterococcus faecalis* encodes two additional SEDS homologs, whose expression is induced in response to antibiotic-mediated cell wall stress by the CroS/R two-component system. However, nothing was previously known about the function of these SEDS homologs. In this work, we determined that these two additional SEDS homologs in *E. faecalis* each possess glycosyltransferase activity *in vitro*, preferentially associate with distinct bPBPs in *E. faecalis*, can functionally substitute for either FtsW or RodA (but not both), and are upregulated in a CroR-dependent manner in response to FtsW depletion, enhancing peptidoglycan synthesis and cephalosporin resistance.

**IMPORTANCE:**

SEDS (shape, elongation, division, and sporulation) proteins are transmembrane glycosyltransferases that play a critical role in synthesis of bacterial peptidoglycan. It is well known that most bacteria possess two SEDS protein homologs, known as FtsW and RodA, that participate in peptidoglycan synthesis at distinct locations in the cell. Some bacterial genomes also encode, in addition to FtsW and RodA, additional SEDS protein homologs whose functions are typically poorly characterized. *Enterococcus faecalis* is a commensal of the human intestinal tract and an important opportunistic pathogen that encodes two such additional SEDS proteins, whose functions have not been reported previously. Our results reveal new insights into the activity and function of these additional SEDS homologs, showing that they are genuine glycosyltransferases that enhance peptidoglycan synthesis and cephalosporin resistance in response to cell wall stress.

## INTRODUCTION

*Enterococcus faecalis* and *Enterococcus faecium*, commensals of the human gastrointestinal tract, are considered opportunistic pathogens and can cause serious hospital-acquired infections, such as bacteremia, endocarditis, urinary tract infections, and surgical site infections (1, 2). These enterococcal infections can be life-threatening because enterococci possess several mechanisms of intrinsic and acquired resistance to numerous antimicrobial agents, including nearly all of the current therapies used to treat enterococcal infections (3, 4), stressing the urgent demand for novel therapeutic strategies to treat or prevent enterococcal infections.

Prior cephalosporin treatment is a major risk factor for developing enterococcal infections due to the intrinsic resistance of enterococci to cephalosporins (5–7). Depletion of cephalosporin-susceptible bacteria in the gut upon cephalosporin treatment enables overgrowth of enterococci, leading to escape from the intestine and infections elsewhere (8–10). One approach to prevent these infections could be to provide an additional therapeutic in combination with cephalosporins to disable cephalosporin resistance and limit enterococcal overgrowth. Therefore, understanding the mechanisms of cephalosporin resistance in clinically relevant enterococci could provide essential insights enabling development of novel drugs.

Cephalosporins inhibit penicillin binding proteins (PBPs), which possess transpeptidase (TPase) activity that catalyzes the crosslinking of peptidoglycan (11, 12). Class B PBPs (bPBPs) are thought to form specific, cognate pairs with multi-pass transmembrane SEDS (shape, elongation, division, and sporulation) proteins, which possess glycosyltransferase (GTase) activity and polymerize peptidoglycan strands. The bPBP-SEDS complexes thereby create “peptidoglycan synthases” that perform both GTase and TPase activity (13–17). Within these synthase complexes, the bPBP activates the GTase activity of the partner SEDS protein (13–15, 18). Many species of bacteria encode two SEDS proteins, called FtsW and RodA, which each form the core of two distinct peptidoglycan synthases functioning at different locations in the cell (19–21). We previously demonstrated that the bPBPs PbpB and PbpA preferentially co-purify with and specifically activate the GTase activity of FtsW and RodA, respectively, in *E. faecalis* (18).

The *E. faecalis* genome encodes three bPBPs (PbpB, PbpA, Pbp4) that are each individually required for cephalosporin resistance (18, 22). Pbp4 and PbpA exhibit low reactivity with cephalosporins and therefore are not inactivated during cephalosporin exposure (22–24). The TPase activity of PbpB is readily inactivated by cephalosporins, but PbpB is nevertheless required for cephalosporin resistance because it specifically activates the GTase activity of FtsW, which is also required for cephalosporin resistance in *E. faecalis* (18). Hence, the coordinated action of peptidoglycan synthases is required for enterococci to resist cephalosporin stress. The genome of *E. faecalis* encodes four SEDS protein homologs (FtsW, RodA, OG1RF_11070, and OG1RF_11071), although nothing was previously known about the function of the latter two.

We previously found that OG1RF_11070 and OG1RF_11071 were upregulated in response to activation of the CroS/R two-component signaling system in *E. faecalis* (25). CroS, the sensor kinase, is activated in response to antibiotic-mediated cell wall stress (e.g., exposure to various antibiotics that inhibit peptidoglycan synthesis). Activated CroS phosphorylates the response regulator, CroR, which binds DNA to regulate gene expression (26–28). The CroS/R system is required for resistance to several cell wall-targeting antibiotics, including cephalosporins (28). The CroR regulon has been defined in *E. faecalis* and includes the two additional SEDS proteins OG1RF_11070 and OG1RF_11071 (25), suggesting that these SEDS proteins may play a role in peptidoglycan synthesis during cell wall stress. However, nothing was previously known about the role of CroS/R-dependent regulation of OG1RF_11070 and OG1RF_11071 in the cell wall stress response of *E. faecalis*.

Here, we report the first investigation of the function of the *E. faecalis* SEDS proteins OG1RF_11070 and OG1RF_11071. We demonstrated that both OG1RF_11070 and OG1RF_11071 possess GTase activity *in vitro*, preferentially co-purify with distinct bPBP partners, and can each functionally substitute for either FtsW or RodA (but not both) and that CroR-dependent upregulation enhances peptidoglycan synthesis and cephalosporin resistance upon depletion of FtsW.

## RESULTS

### OG1RF_11070 and OG1RF_11071 are similar to FtsW and RodA, respectively

**Fig 1:**
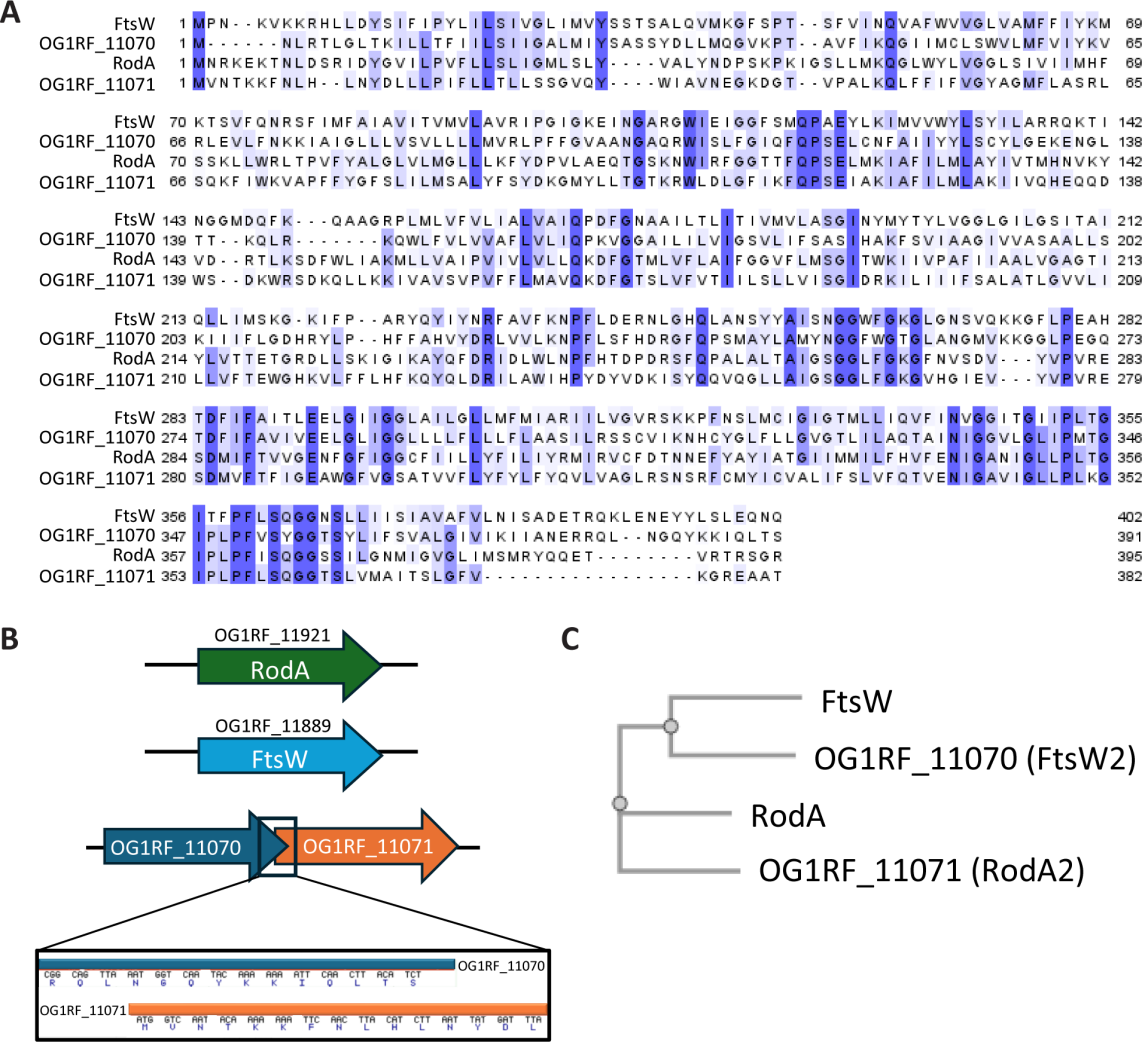
OG1RF_11070 and OG1RF_11071 resemble FtsW and RodA, respectively. (**A**) A multiple sequence alignment of the four SEDS proteins from *E. faecalis* OG1RF (FtsW = OG1RF_11889; FtsW2 = OG1RF_11070; RodA = OG1RF_11921; RodA2 = OG1RF_11071) was performed via Multiple Alignment using Fast Fourier Transform (MAFFT) from the European Molecular Biology Laboratory-European Bioinformatics Institute (EMBL-EBI). The blue color indicates percent identity between sequences, with darker blue representing a higher percentage of the sequences that contain the identical amino acid. (**B**) Genetic organization of the four SEDS genes in *E. faecalis* OG1RF. (**C**) A phylogenetic tree was created via Simple Phylogeny from EMBL-EBI using the multiple sequence alignment shown in panel **A**.

Comparison of the amino acid sequences of the four SEDS proteins (Fig. 1A) revealed conservation among all four homologs of a key aspartate residue (D284 in FtsW), which is required for glycosyltransferase activity of SEDS homologs (15). The phylogenetic relationship inferred from the sequence alignment indicated that OG1RF_11070 was more closely related to FtsW than OG1RF_11071 or RodA, and OG1RF_11071 was more closely related to RodA than to FtsW or OG1RF_11070. Hence, despite the fact that OG1RF_11070 and OG1RF_11071 are encoded adjacent to each other on the chromosome, the phylogenetic analysis suggests that they did not result from a gene duplication (Fig. 1B and C). Based on the sequence analysis and functional data (see below), we renamed OG1RF_11070 as FtsW2 and OG1RF_11071 as RodA2.

**Fig 2:**
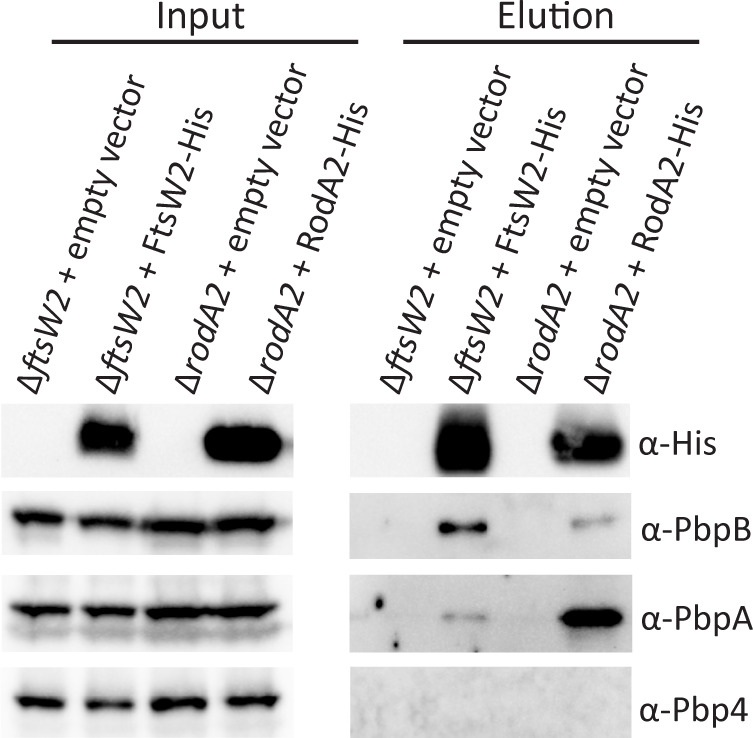
FtsW2 and RodA2 preferentially associate with PbpB and PbpA, respectively, in *E. faecalis*. FtsW2-His or RodA2-His was enriched after formaldehyde crosslinking from exponentially growing *E. faecalis* cells using immobilized metal affinity chromatography. Crosslinks were reversed, and input (lysate) and elution fractions were subjected to SDS-PAGE and immunoblotting with antisera to detect His, PbpB, PbpA, or Pbp4. Data are representative of two individual experiments. Strains were Δ*ftsW2* + empty vector = MN31 (pJRG9); Δ*ftsW2* + FtsW2-His = MN31 (pMEL32); Δ*rodA2* + empty vector = MN32 (pJRG9); Δ*rodA2* + RodA2-His = MN32 (pMEL33).

Each SEDS protein is thought to associate physically and functionally with a specific cognate bPBP (13–17). In *E. faecalis*, we previously demonstrated that FtsW preferentially associates with PbpB, and RodA preferentially associates with PbpA (18). Therefore, we probed for *in vivo* associations between FtsW2 or RodA2 and bPBPs by enriching for His-tagged FtsW2 or RodA2 from *E. faecalis* cells and assessing co-purification of bPBPs. Both FtsW2-His and RodA2-His were effectively enriched from cell lysates (Fig. 2, αHis). FtsW2 co-purified more efficiently with PbpB than PbpA, and RodA2 co-purified more efficiently with PbpA than PbpB (Fig. 2), similar to the previous observations for FtsW and RodA, respectively (18). No associations between the other bPBP in *E. faecalis*, Pbp4, and FtsW2 or RodA2 were detected under these conditions (Fig. 2).

Expression levels for the four SEDS homologs in *E. faecalis* were analyzed by RT-qPCR (because antibodies to detect the SEDS proteins are not available). *ftsW2* and *rodA2* exhibited relatively low levels of expression compared to *ftsW* and *rodA* in wild-type cells grown under standard laboratory conditions (Fig. S1), suggesting that FtsW and RodA are the primary contributors to peptidoglycan synthesis during growth in the absence of exogenous stress. In agreement, deletion of *ftsW2* or *rodA2* individually or together from the chromosome (Δ*ftsW2,* Δ*rodA2,* or ∆(*ftsW2 rodA2*) strains) had no effect on growth compared to WT cells (Fig. S2), demonstrating that FtsW2 and RodA2 are not required for growth in standard laboratory conditions.

### FtsW2 and RodA2 can enhance cephalosporin resistance in a mutant-specific manner

We previously showed that FtsW is required for cephalosporin resistance in *E. faecalis* (18), and the requirement of RodA had not been tested. To analyze the requirement of RodA and the other two *E. faecalis* SEDS proteins for cephalosporin resistance, the minimal inhibitory concentration (MIC) of ceftriaxone, a representative cephalosporin, was measured in *E. faecalis* strains with deletion or depletion of the various SEDS proteins. Initially, we constructed an *E. faecalis* strain with a deletion of *rodA*; however, this deletion mutant exhibited a severe growth defect, and suppressors with altered growth properties emerged readily. As a result, we instead constructed a RodA depletion strain (denoted “dep-*rodA*”), in which RodA was expressed from a plasmid-borne nitrate-inducible promoter while chromosomal *rodA* was deleted, using an approach that we previously described (29) and that we used to make a FtsW depletion strain (18). When cultured in the absence of inducer, depletion of *rodA* or *ftsW* transcripts was verified by RT-qPCR in the corresponding depletion strain (Fig. S3).

Although we previously found that depletion of FtsW using this approach resulted in a growth defect (18), no growth defect was observed upon depletion of RodA (Fig. S4). Because the *rodA* deletion mutant exhibited a severe growth defect, this result suggests that residual (i.e., leaky) *rodA* expression in the dep-*rodA* strain cultured without inducer (Fig. S3) was sufficient to support growth in the absence of exogenous stress. However, depletion of RodA resulted in reduced cephalosporin resistance, as demonstrated by a decrease in the MIC compared to WT cells (Table 1), identifying RodA as a novel determinant for cephalosporin resistance in enterococci. In contrast, deletion mutants lacking either *ftsW2,* or *rodA2*, or both all exhibited no differences in resistance (Table 1). Thus, it appears that neither FtsW2 nor RodA2 is important for cephalosporin resistance in otherwise wild-type cells. We also tested for defects of the ∆(*ftsW2 rodA2*) mutant in resistance to other stresses including vancomycin, SDS, lysozyme, chlorhexidine, bacitracin, ampicillin, or cefepime, but no defects were observed (Table S1).

**TABLE 1:** FtsW2 and RodA2 enhance cephalosporin resistance in a mutant-dependent manner

Strain + plasmid^*b*^	MIC _cef_ (µg/mL)^*a*^
WT	64
∆*ftsW2*	64
∆*rodA2*	64
∆(*ftsW2 rodA2*)	64
WT + empty vectors	64
dep-*rodA* + empty vector	8
dep-*rodA* + RodA	128
dep-*rodA* + FtsW	4
dep-*rodA* + FtsW2	8
dep-*rodA* + RodA2	32
dep-*ftsW* + empty	≤1
dep-*ftsW* + RodA	≤1
dep-*ftsW* + FtsW	32
dep-*ftsW* + FtsW2	64
dep-*ftsW* + RodA2	≤1

^*a*^ The median minimal inhibitory concentration (MIC) of ceftriaxone (cef) was determined in the absence of NaNO_3_ from at least three biological replicates.

^*b*^ Strains were WT (wild-type) = OG1; ∆*ftsW2 *= MN31; ∆*rodA2 *= MN32; ∆(*ftsW2 rodA2*) = JL529; WT + empty vectors = OG1 (pJLL286) (pJRG9); dep-*rodA* + empty vector = MN34 (pMEN74) (pJRG9); dep-*rodA* + RodA = MN34 (pMEN74) (pMEL29); dep-*rodA* + FtsW = MN34 (pMEN74) (pMEL31); dep-*rodA* + FtsW2 = MN34 (pMEN74) (pMEL32); dep-*rodA* + RodA2 = MN34 (pMEN74) (pMEL33); dep-*ftsW *+ empty = ML2 (pMEL34) (pJRG9); dep-*ftsW *+ RodA = ML2 (pMEL34) (pMEL29); dep-*ftsW *+ FtsW = ML2 (pMEL34) (pMEL31); dep-*ftsW *+ FtsW2 = ML2 (pMEL34) (pMEL32); dep-*ftsW *+ RodA2 = ML2 (pMEL34) (pMEL33).

To test whether overexpression of FtsW2 or RodA2 could rescue cephalosporin resistance defects of the FtsW or RodA depletion strains, we expressed FtsW2 or RodA2 from a plasmid-borne constitutive promoter in the dep-*ftsW* and dep-*rodA* depletion strains and evaluated ceftriaxone resistance under conditions where FtsW or RodA were depleted. Overexpression of FtsW2 (but not RodA or RodA2) enhanced ceftriaxone resistance upon depletion of FtsW, while overexpression of RodA2 (but not FtsW or FtsW2) enhanced ceftriaxone resistance upon depletion of RodA (Table 1). Hence, these results support the analysis of sequence similarity described above: FtsW2 is more closely related to FtsW and can functionally substitute for FtsW (but not RodA), while RodA2 is more closely related to RodA and can functionally substitute for RodA (but not FtsW). These results are consistent with the co-purification results described above and indicate not only that FtsW2 and RodA2 can functionally substitute for their respective SEDS homolog (FtsW or RodA) under some circumstances but also that FtsW2 and RodA2 are functionally distinct from each other.

### FtsW2 and RodA2 possess glycosyltransferase (GTase) activity

**Fig 3:**
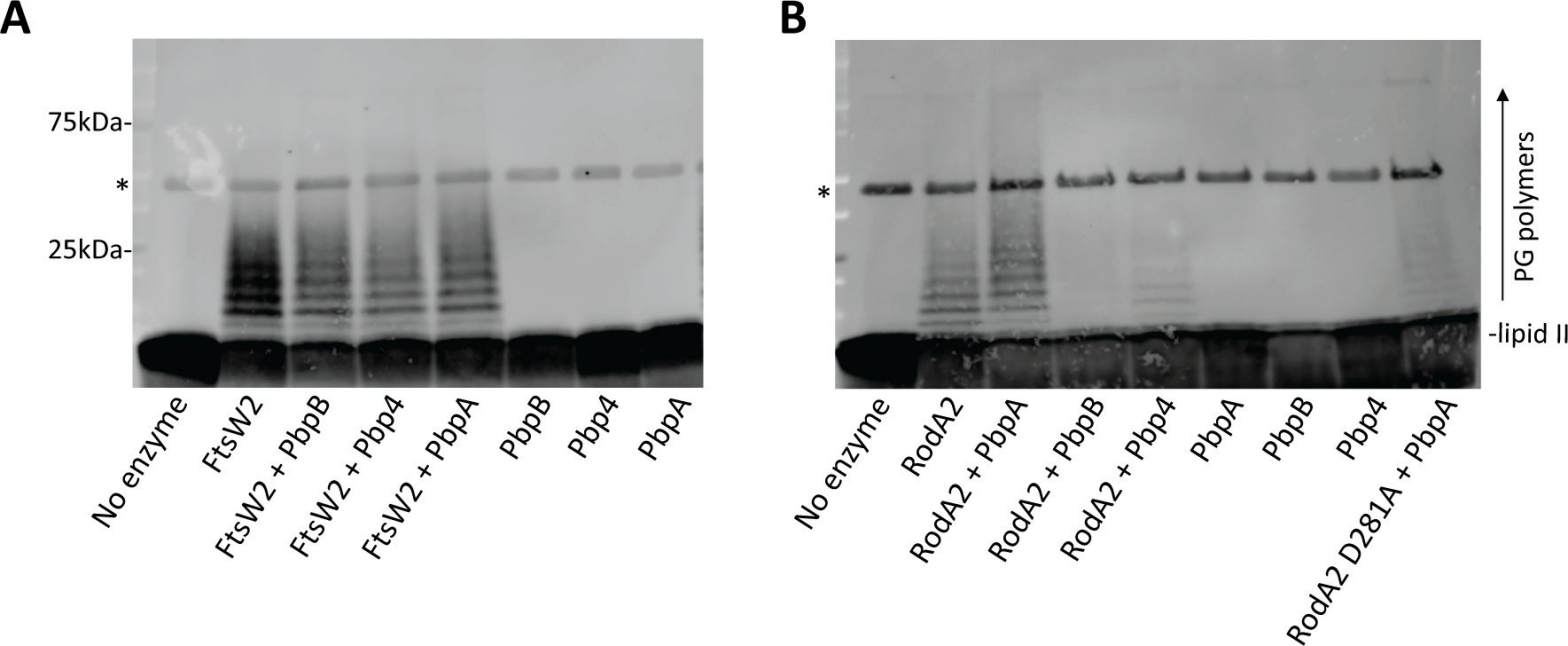
FtsW2 and RodA2 possess glycosyltransferase activity. *E. faecalis* lipid II was incubated with FtsW2-His (**A**) or RodA2-His (**B**) in the presence or absence of bPBPs to allow for polymerization of peptidoglycan strands. The stem peptide of lipid II or polymerized peptidoglycan (PG) was biotinylated for detection after SDS-PAGE using streptavidin-conjugated dye. Proteins included in each reaction are noted below the lanes. “No enzyme” represents a reaction with no SEDS protein or bPBP added. The band marked by * in all lanes is PbpX (used to biotinylate PG polymers). Data are representative of at least two independent experiments.

SEDS proteins are GTases involved in peptidoglycan synthesis, and the cognate bPBP typically activates the GTase activity of the corresponding SEDS protein (5, 6). We previously demonstrated that PbpB and PbpA activate the GTase activity of FtsW and RodA, respectively, in *E. faecalis* (18). To determine if FtsW2 and RodA2 also possess GTase activity and probe for any potential regulation by the bPBPs, we performed *in vitro* GTase activity assays, as previously described (14, 15, 30, 31), with purified FtsW2 or RodA2 in the presence or absence of bPBPs. Briefly, purified SEDS proteins, with or without addition of purified bPBPs, were incubated with lipid II, the peptidoglycan precursor and SEDS GTase substrate, to allow polymerization of peptidoglycan strands. The resulting peptidoglycan polymers (along with unpolymerized lipid II) were then biotinylated for detection after SDS-PAGE using a streptavidin-conjugated dye. As expected, no polymerization was present in reactions containing only PbpB, PbpA, or Pbp4, in the absence of a SEDS protein (Fig. 3A and B). FtsW2 exhibited relatively robust GTase activity without the addition of any bPBP, as demonstrated by the appearance of higher molecular weight bands corresponding to polymerized peptidoglycan. Moreover, the GTase activity of FtsW2 was not further enhanced by the addition of any of the bPBPs (Fig. 3A). RodA2 also exhibited GTase activity in the absence of bPBPs, and RodA2 activity appeared to be modestly enhanced by the addition of PbpA (increased abundance of higher molecular weight peptidoglycan polymer bands) but not by the addition of PbpB or Pbp4 (Fig. 3B). In fact, the addition of PbpB or Pbp4 seemed to impair the GTase activity of RodA2 to some extent, although it is unknown if this decrease in polymerization is of biological relevance. To verify that the observed GTase activity was due to RodA2 and not any residual contaminating co-purifying GTase, we mutated a conserved aspartic acid residue in RodA2 (D281), shown to be important for GTase activity in other SEDS homologs (13, 15, 32), to alanine. GTase activity of the purified RodA2 D281A mutant was then assessed. Polymerization of peptidoglycan was greatly reduced in reactions with RodA2 D281A + PbpA compared to wild-type RodA2 + PbpA, indicating that the peptidoglycan polymerization observed is due to RodA2 activity (Fig. 3B). We attempted to perform a similar experiment with the analogous catalytically impaired FtsW2 alanine substitution mutant, but expression of FtsW2 D275A resulted in a substantial growth defect, which prevented us from purifying the mutant protein.

### *ftsW2* and *rodA2* are upregulated in response to depletion of FtsW in a CroR-dependent manner

**Fig 4:**
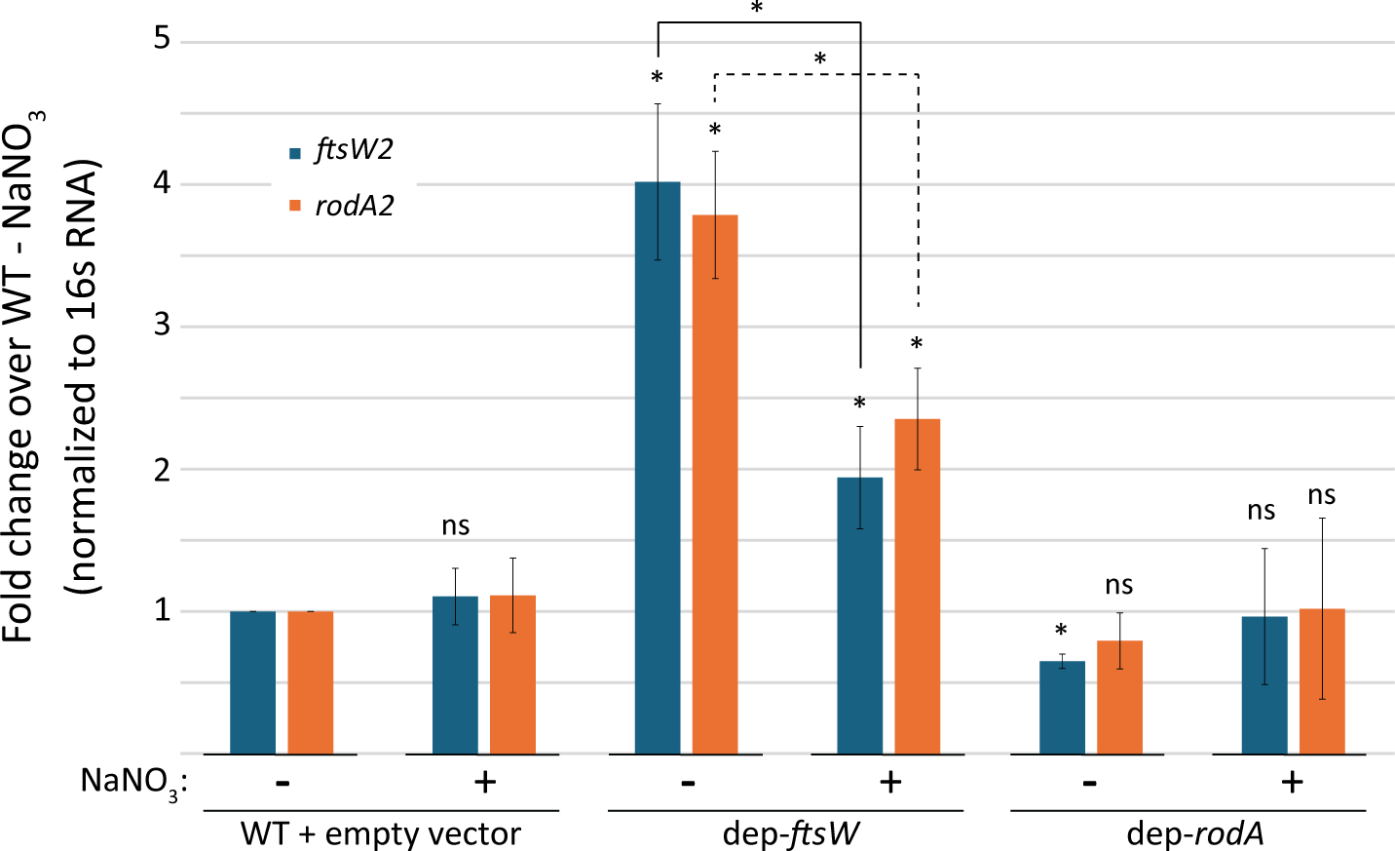
Depletion of FtsW leads to an increase in *ftsW2* and *rodA2* transcript. *ftsW2* and *rodA2* transcript levels from exponentially growing *E. faecalis* cells in the presence or absence of 25 mM NaNO_3_ (inducer) were determined by RT-qPCR. Data represent the mean ± standard deviation of three biological replicates analyzed in technical triplicate. Statistical analysis above each bar graph is compared to WT - NaNO_3_, and additional comparisons are indicated; ns, not statistically significant (*P*  ≥  0.05) and *P <  0.05, as determined by an unpaired two-tailed parametric t test. Strains were WT = OG1 (pJLL286); dep-*rodA* = MN34 (pMEN74); dep-*ftsW* = ML2 (pMEL34).

We previously reported that depletion of FtsW resulted in a growth defect and aberrant cell morphology (18), indicative of a problem with the peptidoglycan cell wall, which would be expected upon depletion of a key peptidoglycan synthase. We also found that FtsW2 and RodA2 are upregulated in response to antimicrobial-mediated cell wall stress by the CroS/R two-component system (25), suggesting the hypothesis that expression of FtsW2 and RodA2 might be upregulated in response to cell wall stress during FtsW depletion (or potentially RodA as well). To quantify expression of FtsW2 and RodA2, we analyzed *ftsW2* and *rodA2* transcript levels by RT-qPCR upon FtsW or RodA depletion compared to WT. In *E. faecalis* dep-*ftsW* cells cultured in the absence of inducer (i.e., upon depletion of FtsW), *ftsW2* and *rodA2* transcript levels were about fourfold higher than in WT cells, and this increase was partially ameliorated with the addition of inducer to enhance FtsW expression (Fig. 4). In contrast, upon depletion of RodA, *ftsW2* and *rodA2* transcript levels remained unchanged compared to WT (Fig. 4), which could be due to leaky expression of RodA in the absence of inducer, consistent with the lack of a growth phenotype (Fig. S3 and S4). This suggests that the dep-*rodA* cells are not experiencing sufficient cell wall stress to trigger enhanced expression of *ftsW2* and *rodA2*.

To assess if the upregulation of *ftsW2* and *rodA2* in response to depletion of FtsW was due to CroR activation, we first evaluated the levels of *croR* transcript upon depletion of FtsW compared to WT cells, because CroS/R upregulates its own expression upon activation (26–28). *croR* expression increased upon depletion of FtsW, and this increase was partially ameliorated upon addition of the inducer to increase FtsW expression (Fig. S5), indicating activation of CroS/R signaling upon FtsW depletion.

**Fig 5:**
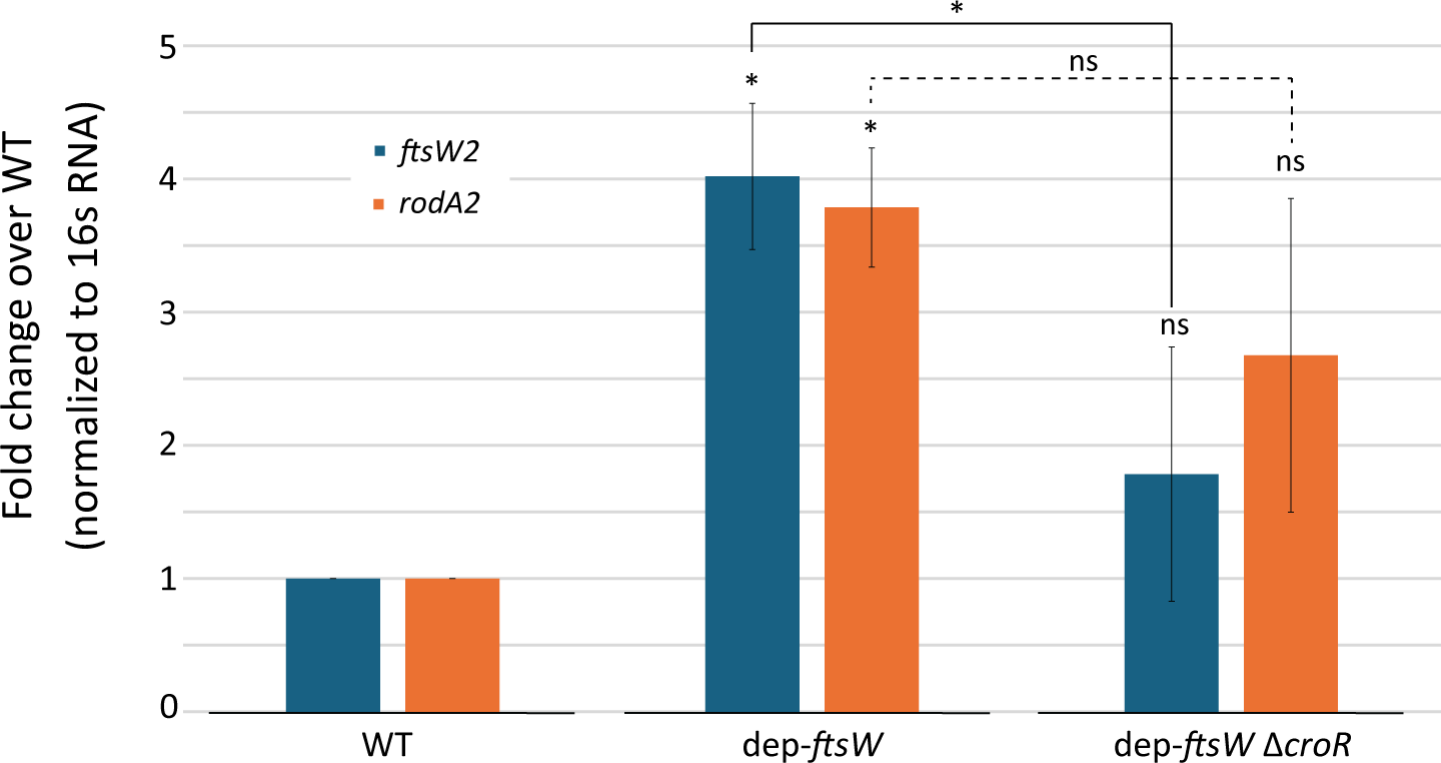
CroR is required for full induction of *ftsW2* or *rodA2* transcript levels upon depletion of FtsW**.** *ftsW2* and *rodA2* transcript levels from exponentially growing *E. faecalis* cells were determined by RT-qPCR. Data represent the mean ± standard deviation of three biological replicates analyzed in technical triplicate. Statistical analysis above each bar graph is compared to WT - NaNO_3_, and additional comparisons are indicated; ns, not statistically significant (*P*  ≥  0.05) and *P <  0.05, as determined by an unpaired two-tailed parametric t test. Strains were WT = OG1 (pJLL286); dep-*ftsW* = ML2 (pMEL34); dep-*ftsW* Δ*croR* = MN38 (pMEL34).

To determine if the upregulation of *ftsW2* and *rodA2* upon depletion of FtsW was specifically due to CroS/R signaling, we constructed the FtsW depletion in a strain lacking *croR,* resulting in dep-*ftsW* Δ*croR,* and assessed *ftsW2* and *rodA2* transcript levels. Upon FtsW depletion in the absence of *croR*, the increase in *ftsW2* and *rodA2* transcript levels was reduced compared to cells depleted of FtsW in the presence of CroR and was not significantly different than WT cells. In other words, the level of *ftsW2* transcript was significantly reduced upon depletion of FtsW in the absence of *croR*, compared to its level in the presence of *croR* (Fig. 5). These results indicate that the increase in *ftsW2* transcript upon depletion of FtsW requires signaling through CroS/R, at least in part.

### CroR-dependent upregulation of *ftsW2* and *rodA2* upon FtsW depletion enhances peptidoglycan synthesis and cephalosporin resistance

**Fig 6:**
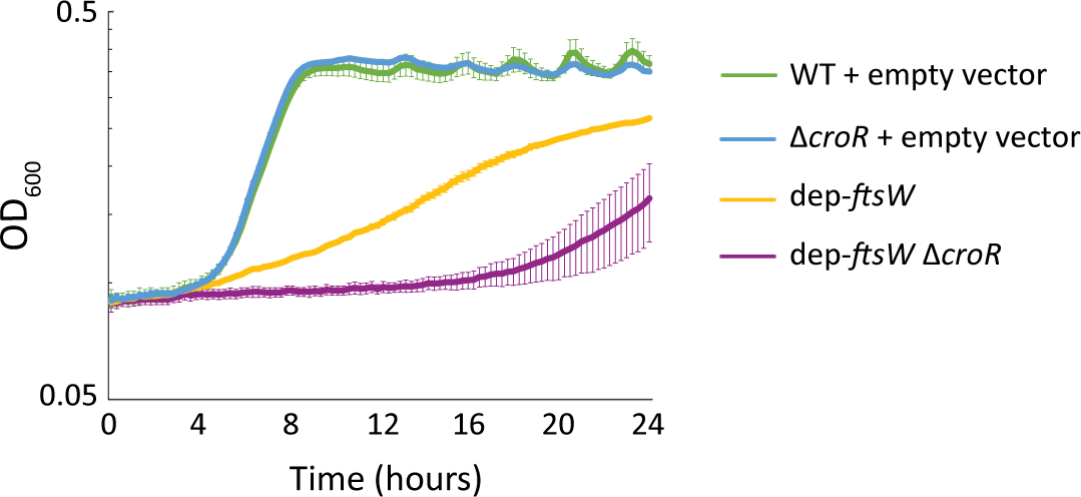
CroR enhances growth upon depletion of FtsW. Growth of *E. faecalis* mutant strains lacking *croR*, depleted of FtsW, or both compared to WT in the absence of inducer. The OD_600_ of normalized cultures was determined every 15 min for 24 h using a Bioscreen C plate reader. Data represent the mean ± standard deviation of three biological replicates. Strains were wild type (WT) + empty vector = OG1 (pJLL286); Δ*croR* + empty vector = SB23 (pJLL286); dep-*ftsW* = ML2 (pMEL34); dep-*ftsW* Δ*croR* = MN38 (pMEL34).

To determine if CroR-dependent upregulation of *ftsW2* helps the cell adapt upon depletion of FtsW, we analyzed growth kinetics and resistance to ceftriaxone. Upon FtsW depletion, the dep-*ftsW* Δ*croR* strain exhibited a more substantial growth defect and reduced ceftriaxone resistance compared to the FtsW depletion strain (Fig. 6, Table 2). These results support the hypothesis that activated CroR upregulates *ftsW2* to help compensate for FtsW depletion.

**TABLE 2:** CroR enhances ceftriaxone resistance upon depletion of FtsW

Strain + plasmid^*b*^	MIC _cef_ (µg/mL)^*a*^	
	- NaNO_3_	+ NaNO_3_
WT + empty vector	64	128
Δ*croR* + empty vector	4	4
dep-*ftsW*	0.25	4
dep-*ftsW* Δ*croR*	≤0.125	1

^*a*^ The median minimal inhibitory concentration (MIC) of ceftriaxone (cef) was determined from at least three biological replicates.

^*b*^ Strains were WT + empty vector = OG1 (pJLL286); Δ*croR* + empty vector = SB23 (pJLL286); dep-*ftsW *= ML2 (pMEL34); dep-*ftsW *Δ*croR *= MN38 (pMEL34).

**Fig 7:**
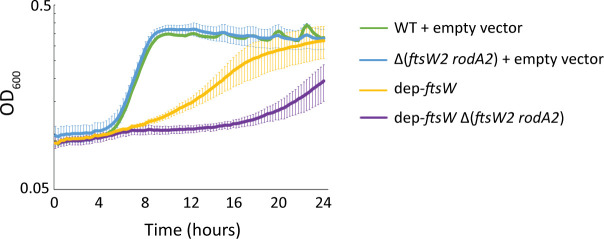
Expression of the *ftsW2* and *rodA2* genes enhances growth upon depletion of FtsW. Growth of *E. faecalis* mutant strains lacking *ftsW2-rodA2*, depleted of FtsW, or both compared to wild type (WT). Data represent the mean ± standard deviation of three biological replicates. Strains were WT + empty vector = OG1 (pJLL286); Δ(*ftsW2 rodA2*) + empty vector = JL529 (pJLL286); dep-*ftsW* = ML2 (pMEL34); dep-*ftsW* Δ(*ftsW2 rodA2*) = MN37 (pMEL34).

To determine whether the CroR-dependent enhancement of growth and ceftriaxone resistance was specifically due to the upregulation of *ftsW2* and *rodA2* (rather than any other CroR-dependent genes), we constructed the FtsW depletion in a strain lacking *ftsW2* and *rodA2,* resulting in dep-*ftsW* ∆(*ftsW2 rodA2*), and analyzed growth kinetics and cephalosporin resistance. Similar to cells lacking *croR* (Fig. 6), there was a more substantial growth defect when FtsW was depleted in cells lacking *ftsW2* and *rodA2*, compared to FtsW depletion alone (Fig. 7). Additionally, FtsW depletion in the absence of *ftsW2* and *rodA2* resulted in a modest but reproducible decrease in cephalosporin resistance compared to depletion of FtsW alone (Table 3). Thus, these results support the hypothesis that CroR-dependent upregulation of FtsW2 is important to help the cell compensate for depletion of FtsW.

**TABLE 3:** Expression of the *ftsW2* and *rodA2* genes enhances ceftriaxone resistance upon depletion of FtsW

Strain + plasmid^*b*^	MIC _cef_ (µg/mL)^*a*^	
	- NaNO_3_	+ NaNO_3_
WT + empty vector	128	128
Δ(*ftsW2 rodA2*) + empty vector	64	128
dep-*ftsW*	0.25	4
dep-*ftsW* Δ(*ftsW2 rodA2*)	0.125	2

^*a*^ The median minimal inhibitory concentration (MIC) of ceftriaxone (cef) was determined from at least three biological replicates.

^*b*^ Strains were WT + empty vector = OG1 (pJLL286); Δ(*ftsW2 rodA2*) + empty vector = JL529 (pJLL286); dep-*ftsW *= ML2 (pMEL34); dep-*ftsW *Δ(*ftsW2 rodA2*) = MN37 (pMEL34).

**Fig 8:**
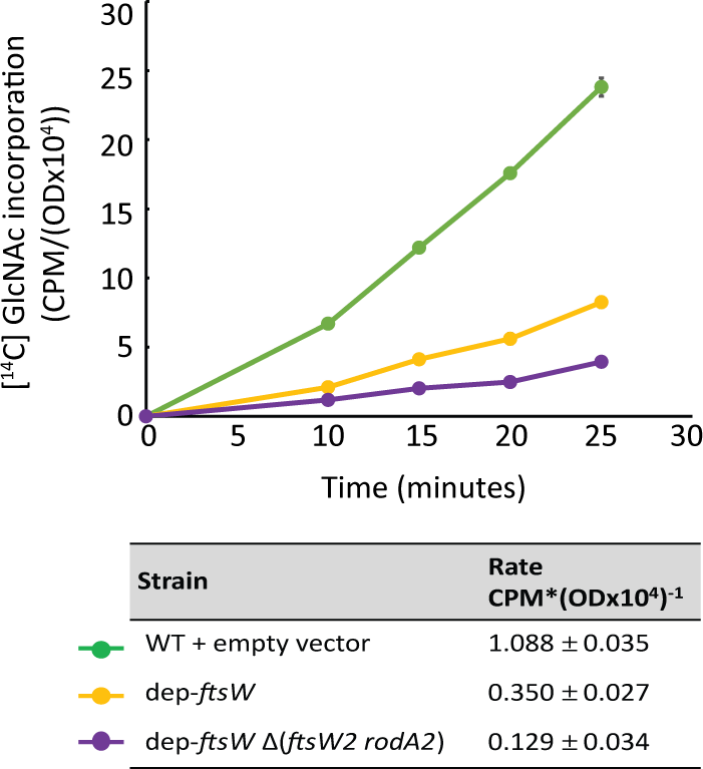
Expression of the *ftsW2* and *rodA2* genes enhances peptidoglycan synthesis upon depletion of FtsW. Exponentially growing *E. faecalis* strains were pulse-labeled with [^14^C] GlcNAc, and incorporation of [^14^C] GlcNAc into the SDS-insoluble peptidoglycan was determined (CPM = counts per minute) normalized to growth (OD = optical density). Incorporation rates were determined from data points between 10 and 20 min and are reported in the table. Data represent the mean ± standard deviation of two biological replicates. Strains were WT + empty vector = OG1 (pJLL286); dep-*ftsW* = ML2 (pMEL34); dep-*ftsW* Δ(*ftsW2 rodA2*) = MN37 (pMEL34).

To directly test if upregulation of FtsW2 and RodA2 enhances peptidoglycan synthesis upon FtsW depletion, we measured the rate of peptidoglycan synthesis in the dep-*ftsW* strain compared to dep-*ftsW* ∆(*ftsW2 rodA2*) by monitoring [^14^C] GlcNAc incorporation into peptidoglycan in exponentially growing cells. Depletion of FtsW in the absence of *ftsW2* and *rodA2* resulted in a slower rate of incorporation compared to that in the presence of *ftsW2* and *rodA2* (Fig. 8), indicating that FtsW2 and/or RodA2 enhance peptidoglycan synthesis when FtsW is depleted. Overall, the data are consistent with the model in which activation of signaling through CroS/R upon FtsW depletion leads to CroR-dependent upregulation of *ftsW2* and *rodA2* to enhance peptidoglycan synthesis, thereby helping improve growth and cephalosporin resistance.

## DISCUSSION

SEDS proteins catalyze glycosyltransferase reactions to polymerize peptidoglycan, forming complexes with cognate bPBPs as part of peptidoglycan synthases (13–15, 18). Most bacterial genomes only encode for two SEDS proteins, FtsW and RodA, which form the core of two different peptidoglycan synthases thought to function at distinct locations in the cell. However, a few bacterial genera, including enterococci, encode homologs of not only FtsW and RodA but also additional SEDS proteins. In general, very little is known about the functional significance of these additional SEDS proteins, including whether they even possess GTase activity at all. Here, we determined that the two additional SEDS homologs in *E. faecalis*, FtsW2 and RodA2, each possess GTase activity, associate preferentially with distinct bPBPs, and are upregulated in a CroR-dependent manner in response to FtsW depletion to enhance peptidoglycan synthesis and cephalosporin resistance.

In this work, we demonstrated with purified proteins *in vitro* that both FtsW2 and RodA2 possess GTase activity (Fig. 3). The GTase activity of FtsW2 did not appear to be enhanced by the addition of any bPBPs as potential partners, at least under the conditions of our experiments (Fig. 3), raising the possibility that FtsW2 may not require a cognate bPBP for activation of its GTase activity. However, it seems likely that FtsW2 still coordinates with a bPBP *in vivo* for crosslinking of the peptidoglycan polymerized by FtsW2, as suggested by the preferential co-purification of FtsW2 with PbpB (Fig. 2). Similar to FtsW2, *E. faecalis* RodA2 also exhibited GTase activity in these *in vitro* reactions in the absence of added bPBP partner (Fig. 3), a phenomenon that has been observed previously with other SEDS GTases, including *E. faecalis* RodA (18) and *B. subtilis* RodA (13, 32). Addition of PbpA (but not other bPBPs) appeared to modestly enhance the GTase activity of RodA2 under our conditions (as reflected by increased abundance of longer peptidoglycan polymers; Fig. 3), suggesting that PbpA acts as a cognate partner for RodA2. This is consistent with the observation that RodA2 preferentially co-purified with PbpA from *E. faecalis* cells (Fig. 2) and suggests the possibility that not only does the RodA2-PbpA pair represent a genuine third peptidoglycan synthase complex in *E. faecalis* but also that some bPBPs can functionally associate with more than one SEDS GTase, given that PbpA is known to activate the GTase activity of RodA in *E. faecalis* (18). It is conceivable that PbpA-mediated regulation of RodA2 is more substantial in *E. faecalis* cells than in the simplified *in vitro* reconstituted reactions used here.

Despite the fact that *ftsW2* and *rodA2* are encoded adjacent to each other in the *E. faecalis* genome, sequence analysis revealed that FtsW2 and RodA2 were more closely related to FtsW and RodA, respectively, than to each other (Fig. 1). This suggests that *ftsW2* and *rodA2* likely did not arise from a gene duplication event of one or the other, but rather share a common ancestor with FtsW or RodA, respectively. The genes encoding FtsW2 and RodA2 are widespread among *E. faecalis* genomes. We analyzed the genomes of 17 diverse commensal and clinical *E. faecalis* strains that represent deep nodes in the *E. faecalis* phylogeny (33) and found homologs of *ftsW2* and *rodA2* adjacent to each other in the chromosome of each strain. We also examined several *E. faecium* genomes (1231502, 1231410, Com12) and identified homologs of *ftsW2* but not *rodA2* in each. *L. monocytogenes* also possesses additional SEDS proteins (FtsW2, RodA2, and RodA3). Like *E. faecalis ftsW2* and *rodA2*, *L. monocytogenes ftsW2* and *rodA3* are predicted to be in an operon together, although similar to *E. faecalis L. monocytogenes* FtsW2 more closely resembles *L. monocytogenes* FtsW than RodA3, and RodA3 more closely resembles RodA than FtsW2 (34). Similar to our observations in *E. faecalis* (Fig. S1 and S2), loss of the additional SEDS proteins in *L. monocytogenes* had no impact on growth (34), and *ftsW2* and *rodA3* displayed relatively low basal expression levels in standard laboratory growth conditions (35). This suggests that the additional SEDS proteins function as “backups” for the primary SEDS proteins, FtsW and RodA, with FtsW2 and RodA3 potentially becoming crucial under certain stress conditions when their expression may be increased.

*E. faecalis ftsW2* and *rodA2* were found to be upregulated upon activation of the CroS/R signaling system in response to the cell wall-targeting antibiotics bacitracin or vancomycin (25). In this work, we also demonstrated that *E. faecalis ftsW2* and *rodA2* were upregulated in at least a partially CroR-dependent manner in response to depletion of FtsW (Fig. 4 and 5). *L. monocytogenes ftsW2* and *rodA3* were also identified to be regulon members of a two-component system, CesR/K (36), and their expression was upregulated in response to cell wall targeting agents like ampicillin, penicillin, moenomycin, and cefuroxime (34). However, in contrast to *E. faecalis ftsW2* and *rodA2* (25), *L. monocytogenes ftsW2* and *rodA3* were not upregulated in response to vancomycin treatment (34), indicating some degree of species-specific regulation of the additional SEDS proteins to adapt to diverse environmental conditions.

*E. faecalis* FtsW2 or RodA2 was not required for ceftriaxone resistance in otherwise wild-type *E. faecalis*. However, expression of either FtsW2 or RodA2 enhanced cephalosporin resistance upon depletion of FtsW or RodA in a mutant-specific manner (Table 1), consistent with the hypothesis that FtsW2 and RodA2 can both function as GTases *in vivo*. Moreover, loss of FtsW2 and RodA2 resulted in a growth defect and a reduction in peptidoglycan synthesis when assayed upon depletion of FtsW (Fig. 7 and 8). We did not detect any differences in resistance to vancomycin, SDS, lysozyme, chlorhexidine, bacitracin, ampicillin, or cefepime in the absence of *ftsW2* and *rodA2* compared to WT cells (Table S1 and Table 1). Similarly, simultaneous deletion of *L. monocytogenes ftsW2* and *rodA3* did not affect resistance to a beta-lactam (penicillin) or moenomycin, although a subtle decrease in resistance to cefuroxime, a cephalosporin, was observed with the single deletion of *rodA3* (34). Importantly, our results also identified RodA as a determinant of cephalosporin resistance in *E. faecalis* (Table 3), expanding our repertoire of potential drug targets to disable cephalosporin resistance of enterococci.

Because *E. faecalis ftsW2* and *rodA2* were upregulated upon depletion of FtsW (Fig. 4), this suggests that FtsW2 and/or RodA2 may be important *in vivo* in conditions when FtsW becomes inactivated or unavailable to polymerize peptidoglycan. We are not aware of any naturally occurring inhibitor of SEDS protein GTase activity. However, it seems reasonable to imagine that some microbe or bacteriophage in the biosphere produces an antimicrobial compound that inhibits SEDS protein activity. In addition, there could be some as-yet-unidentified environmental condition, such as altered pH or metal availability, that could lead to the inactivation of FtsW or RodA, thereby resulting in the requirement of FtsW2 or RodA2. Future work will focus on understanding the environmental circumstances in which FtsW2 and RodA2 activity becomes essential, as well as their requirement (or lack thereof) for regulation by bPBP partners in *E. faecalis* cells.

## MATERIALS AND METHODS

### Bacterial strains and growth conditions

*E. coli* strains were grown in lysogeny broth (LB) or agar, or in brain heart infusion (BHI) broth or agar when erythromycin was used for selection. *E. coli* TOP10 and DH5α were used as cloning hosts. Enterococci were grown in Mueller-Hinton broth (MHB) or agar (Difco) at 30°C unless otherwise indicated. For maintenance of plasmids, antibiotics were included when required at the following concentrations: chloramphenicol, 10 μg/mL; kanamycin, 50 μg/mL; ampicillin, 100 μg/mL; erythromycin, 100 μg/mL (*E. coli*) or 10 μg/mL (*E. faecalis*). All cultures were grown aerobically with shaking (225 rpm). Overnight cultures containing NaNO_3_ (inducer for gene expression from pJLL286) were washed two times with MHB to remove any residual NaNO_3_ before diluting the cultures for experiments when NaNO_3_ was omitted. NaNO_3_ was used at a concentration of 25 mM.

### Genetic manipulation of enterococci

*E. faecalis* mutant strains were constructed as previously described (18, 28), using a temperature-sensitive, counter-selectable allelic exchange plasmid (pJH086). Counterselection plates included p-Cl-Phe and 20% sucrose. Upstream and downstream fragments of genomic DNA, surrounding the gene of interest, were amplified and introduced into pJH086 by isothermal assembly (37). Deletion alleles were generated in-frame and included codons at the 5′ and 3′ ends of each gene to avoid disrupting adjacent gene expression. All mutants or complemented strains were constructed independently at least twice and analyzed to verify concordant phenotypes. The inserts of all recombinant plasmids were sequenced in their entirety to ensure the absence of any undesired mutations.

Construction of dep-*rodA*, dep-*ftsW ΔcroR* (Δ*croR* parental strain), and dep-*ftsW Δ(ftsW2 rodA2*) (*Δ(ftsW2 rodA2*) parental strain) strains was performed in cells containing an inducible copy of either *rodA* or *ftsW*, expressed from pJLL286 in the presence of erythromycin and NaNO_3_ to induce expression. For these strains, NaNO_3_ was included in the counterselection plate and in the growth media for all subsequent propagation to express the deleted gene, except as specified for experiments.

### Multiple sequence alignment and phylogenetic tree analysis

The multiple sequence alignment of the four SEDS proteins from *E. faecalis* OG1RF was performed via Multiple Alignment using Fast Fourier Transform (MAFFT) from European Molecular Biology Laboratory-European Bioinformatics Institute (EMBL-EBI) using the default parameters. The phylogenetic tree was then created from the multiple sequence alignment using “Simple Phylogeny” from EMBL-EBI with the default parameters.

### Co-purification of SEDS proteins and bPBPs

FtsW2-His or RodA2-His (and co-purifying proteins) was enriched from exponentially growing cells using immobilized metal affinity chromatography, according to a previously described procedure (18). Briefly, stationary-phase cultures were diluted back to an OD_600_ of 0.01 in MHB with antibiotics for selection when necessary and grown at 37°C to exponential phase (OD_600_ = 0.2). Cells were then treated with 1% formaldehyde for 10 min at room temperature and then quenched by addition of 0.5 M glycine for 5 min at room temperature. Cells were collected by centrifugation and treated with 10 mg/mL lysozyme in lysozyme solution at 37°C for 30 min. Samples were then centrifuged (15,000 rpm for 3 min) and resuspended in binding buffer (50 mM Tris [pH 8.0], 300 mM NaCl, 5 mM imidazole [pH 8.0]). Cells were then subjected to bead beating 30 s on, 30 s off, repeated 12 times at room temperature using a PowerLyzer 24 (Qiagen). The lysates were incubated with 1% SDS to solubilize membrane proteins for 1 h at room temperature with overhead rotation. Samples were then incubated with Ni-charged resin (equilibrated with binding buffer) for 1 h with overhead rotation at room temperature. Samples were then transferred to a Micro Bio-Spin chromatography column (BioRad) and washed with binding buffer. His-tagged proteins were eluted with elution buffer (50 mM Tris [pH 8.0], 300 mM NaCl, 500 mM imidazole [pH 8.0]). Elution samples were concentrated using Amicon Ultra centrifugal filters with a 3KDa cutoff (Millipore Sigma). Laemmli SDS sample buffer was added, and samples were boiled for 20 min to reverse crosslinks. Laemmli SDS sample buffer was added to input and elution samples and boiled for 5 min. Samples were subjected to SDS-PAGE and transferred to polyvinylidene difluoride membranes (PVDF; BioRad or Thermo Fisher Scientific) using a BioRad TurboBlot apparatus. Membranes were blocked with 5% milk and probed with anti-PbpA, anti-PbpB, anti-Pbp4, (custom rabbit polyclonal antisera), or anti-His polyclonal rabbit antisera (Invitrogen). Goat anti-rabbit horseradish peroxidase (HRP)-conjugated secondary antibody (Invitrogen) was used for detection on a BioRad Chemidoc.

### Antimicrobial susceptibility assays and growth curves

Minimal inhibitory concentrations (MIC) determinations and growth curves obtained via a Bioscreen C were performed as previously described (18). Briefly, bacteria from stationary-phase cultures were diluted into wells containing twofold serial dilutions of antibiotics in MHB (including chloramphenicol and erythromycin when required for maintenance of plasmids and NaNO_3_ as indicated) at a normalized density of OD_600_ = 4  ×  10^−5^ (~1  ×  10^5^ CFU for WT). Plates were incubated in a Bioscreen C plate reader at 37°C for 24 h. The OD_600_ was determined every 15 min with brief shaking before each measurement, and the lowest concentration of antibiotic that prevented growth was documented as the MIC. To obtain growth curves, bacteria were processed the same way, and the OD_600_ was determined every 15 min in the absence of ceftriaxone using a Bioscreen C plate reader.

### bPBP purification

His_6_-SUMO-PbpA, His_6_-SUMO-PbpB, or His_6_-SUMO-Pbp4 were purified from *E. coli* Nico21 (DE3) cells, and the His-SUMO tag was removed by Ulp1 protease, as previously described (18).

### SEDS protein purification

FtsW2-His_6_ or RodA2-His_6_ were purified from *E. faecalis* OG1 cells as previously described for SEDS protein purification (18).

### Lipid II extraction and quantification

Lipid II was extracted from *E. faecalis* (OG1) as described by Welsh and co-workers (30). Lipid II was quantified as previously described by Sardis and co-workers (31).

### Preparation of BDL

Biotinylated-D-Lysine was prepared as previously described by Qiao and co-workers (38).

### PbpX purification

Recombinant His_6_-PbpX T36-P429 (EF3129), cloned as reported in the literature (30), was purified from Nico21 (DE3) cells as previously described (18).

### *In vitro* glycosyltransferase activity assay

The *in vitro* glycosyltransferase activity assay was performed as previously described with slight modifications (14, 15, 18, 31). Purified 0.5 µM FtsW2-His_6_ (unless indicated otherwise) and 0.5 µM RodA2-His_6_ were incubated with 1:1 molar ratio of purified PbpB, PbpA, or Pbp4 (unless otherwise indicated), along with 100 µM lipid II in GT reaction buffer (final concentrations of 50 mM Tris [pH 7.4], 20 mM MgCl_2_, 20% DMSO) to a total volume of 20 µL. 0.1% DDM was present in the purified proteins, and therefore, the final concentration of DDM in the reactions was not constant. Reactions were incubated at 25°C for 1 h, quenched by incubation at 98°C for 5 min, and allowed to cool to room temperature. Biotinylated d-lysine (2 mM, working concentration) and purified *E. faecalis* PBPX (10 µM, working concentration) were added to the reactions and incubated at 25°C for 30 min to biotinylate the peptidoglycan. Following the incubation, 26 µL of 2× Laemmli SDS sample buffer was added to quench the reaction. Twenty-two microliters was loaded onto a 4–20% gradient polyacrylamide gel (BioRad), subjected to electrophoresis at 180 V for 35 min, then transferred onto a PVDF membrane (BioRad). The membrane was fixed with 0.4% (vol/vol) paraformaldehyde in PBS for 30 min at room temperature and blocked with SuperBlock blocking buffer (Thermo Fisher Scientific) for 1 h at room temperature. To detect the biotinylated products, the membranes were incubated with IRDye 800CW Streptavidin (LI-COR Biosciences), diluted 1:5,000 in SuperBlock for 1 h at room temperature, washed three times with PBS, and visualized using an Amersham Typhoon Imager (GE Life Sciences).

### RNA extraction

Stationary-phase cultures were diluted to an OD_600_ = 0.01 in MHB with antibiotics for selection when necessary and grown at 37°C to exponential phase (OD_600_ = 0.2 for OG1 and MN34; OD_600_ = 0.1 for ML2 and MN38). Cells were then harvested by an equal volume of 1:1 ethanol: acetone, collected by centrifugation (4,000 rpm for 10 min at 4°C), and washed once with MHB. Samples were then resuspended in 200 µL lysis buffer (10 mM Tris [pH 8.0], 1 mM EDTA, 15 mg/mL lysozyme, 250 U/mL mutanolysin) and incubated for 10 min at 37°C. RNA was then purified using the RNeasy Mini Kit (Qiagen) following the recommended protocol. To remove any contaminating DNA, samples were then treated with Turbo DNA-free Kit (Invitrogen). To concentrate RNA, 1/10 volume of 5M NH_4_Ac (10 µL) was added to the RNA, followed by the addition of 2 volumes of 100% EtOH. RNA was isolated by centrifugation (15 min at 4°C, 13,200 rpm), washed twice with 75% EtOH, and collected by centrifugation (5 min at 4°C, 13,200 rpm) before resuspension in RNase-free H_2_O.

### Analysis of gene expression by quantitative reverse transcription-PCR (RT-qPCR)

RT-qPCR was performed as previously described (25, 27). Briefly, cDNA was synthesized from RNA normalized to 500 ng/µL using SuperScript III First-Strand Synthesis SuperMix (Invitrogen) according to the manufacturer’s instructions. cDNA was then used as template for RT-qPCR using a Bio-Rad iCycler and SsoAdvanced SYBR Green Supermix (Bio-Rad) to obtain amplification and melting curves. Primer efficiencies were determined using known concentrations of *E. faecalis* OG1RF genomic DNA and were within 10% of each other. Relative gene expression (Fig. S1) was calculated using the 2^-ΔCT^ method normalized to 16S rRNA. Fold changes were calculated using the Pfaffl method and 16S rRNA as a reference gene. Technical triplicates of RNA prepared from three biological replicates were performed for gene expression analysis.

## SUPPLEMENTAL MATERIAL

Supplemental material msphere.00528-25-s0001.pdf Supplemental figures and tables.

ASM does not own the copyrights to Supplemental Material that may be linked to, or accessed through, an article. The authors have granted ASM a non-exclusive, world-wide license to publish the Supplemental Material files. Please contact the corresponding author directly for reuse.

## References

[1] Weiner LM, Webb AK, Limbago B, Dudeck MA, Patel J, Kallen AJ, Edwards JR, Sievert DM. 2016. Antimicrobial-resistant pathogens associated with healthcare-associated infections: summary of data reported to the national healthcare safety network at the centers for disease control and prevention, 2011–2014. Infect Control Hosp Epidemiol 37:1288–1301. doi:10.1017/ice.2016.174 27573805 PMC6857725

[2] Magill SS, O’Leary E, Janelle SJ, Thompson DL, Dumyati G, Nadle J, Wilson LE, Kainer MA, Lynfield R, Greissman S, et al.. 2018. Changes in prevalence of health care-associated infections in U.S. hospitals. N Engl J Med 379:1732–1744. doi:10.1056/NEJMoa1801550 30380384 PMC7978499

[3] Kristich CJ, Rice LB, Arias CA. 2014. Enterococcal infection—treatment and antibiotic resistance, p 91–192. In Gilmore M, Clewell D, Ike Y, Shankar N (ed), Enterococci: from commensals to leading causes of drug resistant infection [internet]. Massachusetts Eye and Ear Infirmary, Boston. 24649510

[4] Hollenbeck BL, Rice LB. 2012. Intrinsic and acquired resistance mechanisms in Enterococcus. Virulence 3:421–433. doi:10.4161/viru.21282 23076243 PMC3485979

[5] Murray BE. 1990. The life and times of the Enterococcus. Clin Microbiol Rev 3:46–65. doi:10.1128/CMR.3.1.46 2404568 PMC358140

[6] Shepard BD, Gilmore MS. 2002. Antibiotic-resistant enterococci: the mechanisms and dynamics of drug introduction and resistance. Microbes Infect 4:215–224. doi:10.1016/s1286-4579(01)01530-1 11880055

[7] Carmeli Y, Eliopoulos GM, Samore MH. 2002. Antecedent treatment with different antibiotic agents as a risk factor for vancomycin-resistant Enterococcus. Emerg Infect Dis 8:802–807. doi:10.3201/eid0808.010418 12141965 PMC2732508

[8] Brandl K, Plitas G, Mihu CN, Ubeda C, Jia T, Fleisher M, Schnabl B, DeMatteo RP, Pamer EG. 2008. Vancomycin-resistant enterococci exploit antibiotic-induced innate immune deficits. Nature 455:804–807. doi:10.1038/nature07250 18724361 PMC2663337

[9] Ubeda C, Taur Y, Jenq RR, Equinda MJ, Son T, Samstein M, Viale A, Socci ND, van den Brink MRM, Kamboj M, Pamer EG. 2010. Vancomycin-resistant Enterococcus domination of intestinal microbiota is enabled by antibiotic treatment in mice and precedes bloodstream invasion in humans. J Clin Invest 120:4332–4341. doi:10.1172/JCI43918 21099116 PMC2993598

[10] Donskey CJ, Chowdhry TK, Hecker MT, Hoyen CK, Hanrahan JA, Hujer AM, Hutton-Thomas RA, Whalen CC, Bonomo RA, Rice LB. 2000. Effect of antibiotic therapy on the density of vancomycin-resistant enterococci in the stool of colonized patients. N Engl J Med 343:1925–1932. doi:10.1056/NEJM200012283432604 11136263 PMC4370337

[11] Zapun A, Contreras-Martel C, Vernet T. 2008. Penicillin-binding proteins and beta-lactam resistance. FEMS Microbiol Rev 32:361–385. doi:10.1111/j.1574-6976.2007.00095.x 18248419

[12] Bush K, Bradford PA. 2016. β-lactams and β-lactamase inhibitors: an overview. Cold Spring Harb Perspect Med 6:a025247. doi:10.1101/cshperspect.a025247 27329032 PMC4968164

[13] Meeske AJ, Riley EP, Robins WP, Uehara T, Mekalanos JJ, Kahne D, Walker S, Kruse AC, Bernhardt TG, Rudner DZ. 2016. SEDS proteins are a widespread family of bacterial cell wall polymerases. Nature 537:634–638. doi:10.1038/nature19331 27525505 PMC5161649

[14] Sjodt M, Rohs PDA, Gilman MSA, Erlandson SC, Zheng S, Green AG, Brock KP, Taguchi A, Kahne D, Walker S, Marks DS, Rudner DZ, Bernhardt TG, Kruse AC. 2020. Structural coordination of polymerization and crosslinking by a SEDS-bPBP peptidoglycan synthase complex. Nat Microbiol 5:813–820. doi:10.1038/s41564-020-0687-z 32152588 PMC7540724

[15] Taguchi A, Welsh MA, Marmont LS, Lee W, Sjodt M, Kruse AC, Kahne D, Bernhardt TG, Walker S. 2019. FtsW is a peptidoglycan polymerase that is functional only in complex with its cognate penicillin-binding protein. Nat Microbiol 4:587–594. doi:10.1038/s41564-018-0345-x 30692671 PMC6430707

[16] Emami K, Guyet A, Kawai Y, Devi J, Wu LJ, Allenby N, Daniel RA, Errington J. 2017. RodA as the missing glycosyltransferase in Bacillus subtilis and antibiotic discovery for the peptidoglycan polymerase pathway. Nat Microbiol 2:16253. doi:10.1038/nmicrobiol.2016.253 28085152 PMC5568705

[17] Kumar S, Mollo A, Kahne D, Ruiz N. 2022. The bacterial cell wall: from lipid II flipping to polymerization. Chem Rev 122:8884–8910. doi:10.1021/acs.chemrev.1c00773 35274942 PMC9098691

[18] Nelson ME, Little JL, Kristich CJ. 2024. Pbp4 provides transpeptidase activity to the FtsW-PbpB peptidoglycan synthase to drive cephalosporin resistance in Enterococcus faecalis. Antimicrob Agents Chemother 68:e0055524. doi:10.1128/aac.00555-24 39058024 PMC11373202

[19] Philippe J, Vernet T, Zapun A. 2014. The elongation of ovococci. Microb Drug Resist 20:215–221. doi:10.1089/mdr.2014.0032 24773288 PMC4050454

[20] Briggs NS, Bruce KE, Naskar S, Winkler ME, Roper DI. 2021. The pneumococcal divisome: dynamic control of Streptococcus pneumoniae cell division. Front Microbiol 12:737396. doi:10.3389/fmicb.2021.737396 34737730 PMC8563077

[21] Cameron TA, Margolin W. 2024. Insights into the assembly and regulation of the bacterial divisome. Nat Rev Microbiol 22:33–45. doi:10.1038/s41579-023-00942-x 37524757 PMC11102604

[22] Djorić D, Little JL, Kristich CJ. 2020. Multiple low-reactivity class B penicillin-binding proteins are required for cephalosporin resistance in enterococci. Antimicrob Agents Chemother 64:e02273-19. doi:10.1128/AAC.02273-19 32041714 PMC7179317

[23] Arbeloa A, Segal H, Hugonnet J-E, Josseaume N, Dubost L, Brouard J-P, Gutmann L, Mengin-Lecreulx D, Arthur M. 2004. Role of class A penicillin-binding proteins in PBP5-mediated beta-lactam resistance in Enterococcus faecalis. J Bacteriol 186:1221–1228. doi:10.1128/JB.186.5.1221-1228.2004 14973044 PMC344401

[24] Moon TM, D’Andréa ÉD, Lee CW, Soares A, Jakoncic J, Desbonnet C, Garcia-Solache M, Rice LB, Page R, Peti W. 2018. The structures of penicillin-binding protein 4 (PBP4) and PBP5 from enterococci provide structural insights into β-lactam resistance. J Biol Chem 293:18574–18584. doi:10.1074/jbc.RA118.006052 30355734 PMC6290140

[25] Timmler SB, Kellogg SL, Atkinson SN, Little JL, Djorić D, Kristich CJ. 2022. CroR regulates expression of pbp4(5) to promote cephalosporin resistance in Enterococcus faecalis. mBio 13:e0111922. doi:10.1128/mbio.01119-22 35913163 PMC9426447

[26] Comenge Y, Quintiliani R, Li L, Dubost L, Brouard J-P, Hugonnet J-E, Arthur M. 2003. The CroRS two-component regulatory system is required for intrinsic beta-lactam resistance in Enterococcus faecalis. J Bacteriol 185:7184–7192. doi:10.1128/JB.185.24.7184-7192.2003 14645279 PMC296236

[27] Kellogg SL, Kristich CJ. 2016. Functional dissection of the CroRS two-component system required for resistance to cell wall stressors in Enterococcus faecalis. J Bacteriol 198:1326–1336. doi:10.1128/JB.00995-15 26883822 PMC4859583

[28] Kellogg SL, Little JL, Hoff JS, Kristich CJ. 2017. Requirement of the CroRS two-component system for resistance to cell wall-targeting antimicrobials in Enterococcus faecium. Antimicrob Agents Chemother 61:e02461-16. doi:10.1128/AAC.02461-16 28223383 PMC5404561

[29] Mascari CA, Djorić D, Little JL, Kristich CJ. 2022. Use of an interspecies chimeric receptor for inducible gene expression reveals that metabolic flux through the peptidoglycan biosynthesis pathway is an important driver of cephalosporin resistance in Enterococcus faecalis. J Bacteriol 204:e0060221. doi:10.1128/jb.00602-21 35258319 PMC9017299

[30] Welsh MA, Taguchi A, Schaefer K, Van Tyne D, Lebreton F, Gilmore MS, Kahne D, Walker S. 2017. Identification of a functionally unique family of penicillin-binding proteins. J Am Chem Soc 139:17727–17730. doi:10.1021/jacs.7b10170 29182854 PMC5729098

[31] Sardis MF, Bohrhunter JL, Greene NG, Bernhardt TG. 2021. The LpoA activator is required to stimulate the peptidoglycan polymerase activity of its cognate cell wall synthase PBP1a. Proc Natl Acad Sci USA 118:e2108894118. doi:10.1073/pnas.2108894118 34429361 PMC8536351

[32] Sjodt M, Brock K, Dobihal G, Rohs PDA, Green AG, Hopf TA, Meeske AJ, Srisuknimit V, Kahne D, Walker S, Marks DS, Bernhardt TG, Rudner DZ, Kruse AC. 2018. Structure of the peptidoglycan polymerase RodA resolved by evolutionary coupling analysis. Nature 556:118–121. doi:10.1038/nature25985 29590088 PMC6035859

[33] Palmer KL, Godfrey P, Griggs A, Kos VN, Zucker J, Desjardins C, Cerqueira G, Gevers D, Walker S, Wortman J, Feldgarden M, Haas B, Birren B, Gilmore MS. 2012. Comparative genomics of enterococci: variation in Enterococcus faecalis, clade structure in E. faecium, and defining characteristics of E. gallinarum and E. casseliflavus. mBio 3:e00318-11. doi:10.1128/mBio.00318-11 22354958 PMC3374389

[34] Rismondo J, Halbedel S, Gründling A. 2019. Cell shape and antibiotic resistance are maintained by the activity of multiple FtsW and RodA enzymes in Listeria monocytogenes. mBio 10:e01448-19. doi:10.1128/mBio.01448-19 31387909 PMC6686043

[35] Lobel L, Herskovits AA. 2016. Systems level analyses reveal multiple regulatory activities of CodY controlling metabolism, motility and virulence in Listeria monocytogenes. PLoS Genet 12:e1005870. doi:10.1371/journal.pgen.1005870 26895237 PMC4760761

[36] Nielsen PK, Andersen AZ, Mols M, van der Veen S, Abee T, Kallipolitis BH. 2012. Genome-wide transcriptional profiling of the cell envelope stress response and the role of LisRK and CesRK in Listeria monocytogenes. Microbiology (Reading) 158:963–974. doi:10.1099/mic.0.055467-0 22282521

[37] Gibson DG, Young L, Chuang R-Y, Venter JC, Hutchison CA, Smith HO. 2009. Enzymatic assembly of DNA molecules up to several hundred kilobases. Nat Methods 6:343–345. doi:10.1038/nmeth.1318 19363495

[38] Qiao Y, Lebar MD, Schirner K, Schaefer K, Tsukamoto H, Kahne D, Walker S. 2014. Detection of lipid-linked peptidoglycan precursors by exploiting an unexpected transpeptidase reaction. J Am Chem Soc 136:14678–14681. doi:10.1021/ja508147s 25291014 PMC4210121

