# Supplementary material for: SEDS glycosyltransferases in E. faecalis are upregulated by the CroS/R two-component system to enhance peptidoglycan synthesis during cell wall stress
Source: mSphere. 2025 Nov 24;10(12):e00528-25. doi: 10.1128/msphere.00528-25 (PMC12724132; doi:10.1128/msphere.00528-25)
Supplement: Supplemental material — Supplemental figures and tables. [file msphere.00528-25-s0001.pdf]

Supplemental Material for:

Title: SEDS glycosyltransferases in *E. faecalis* are upregulated by the CroS/R two-component system to enhance peptidoglycan synthesis during cell wall stress

Authors: Madison E Nelson, Dušanka Djorić, Miryah Henriksen-Hadlock, and Christopher J Kristich*

Affiliation:

Department of Microbiology and Immunology

Center for Infectious Disease Research

Medical College of Wisconsin

8701 Watertown Plank Rd

Milwaukee, WI 53226

*to whom correspondence should be addressed: ckristich@mcw.edu

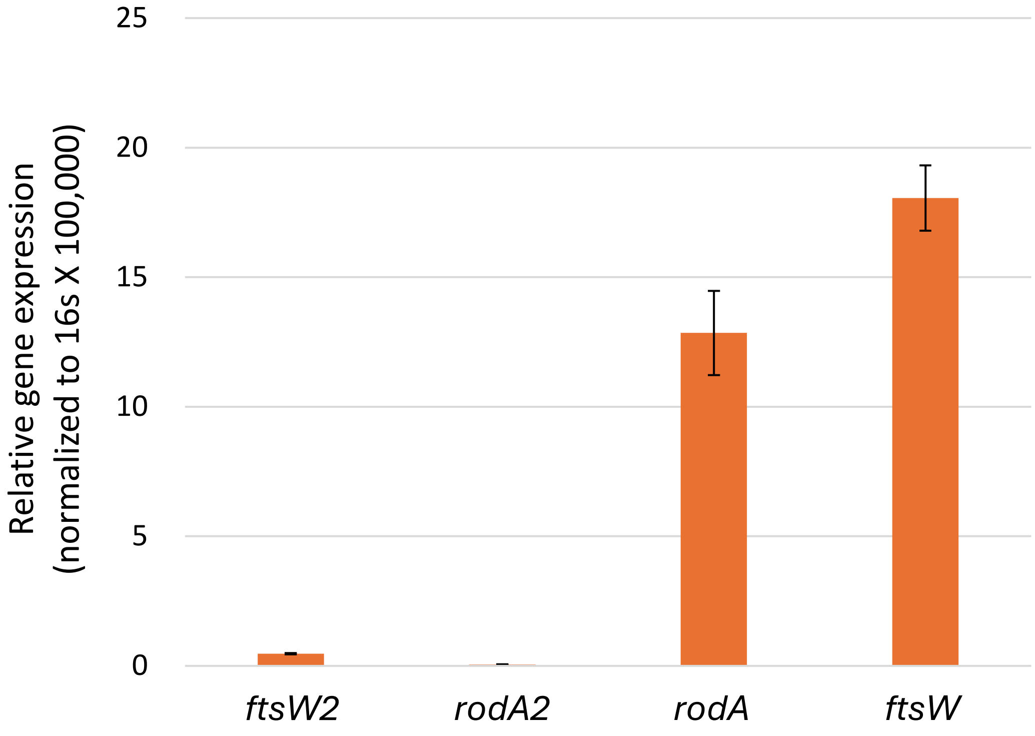

Supplemental Figure 1. *ftsW2* and *rodA2* genes are expressed at relatively low basal levels in wild type *E. faecalis cells*. *ftsW2*, *rodA2*, *ftsW*, or *rodA* transcript levels from exponentially growing *E. faecalis* cells determined by RT-qPCR. Expression levels were normalized to 16s rRNA and multiplied by 100,000. Data represent the mean ± standard deviation of three biological replicates analyzed in technical triplicate. Primer efficiencies were within 10% of each other. The strain used was OG1 (pJLL286).

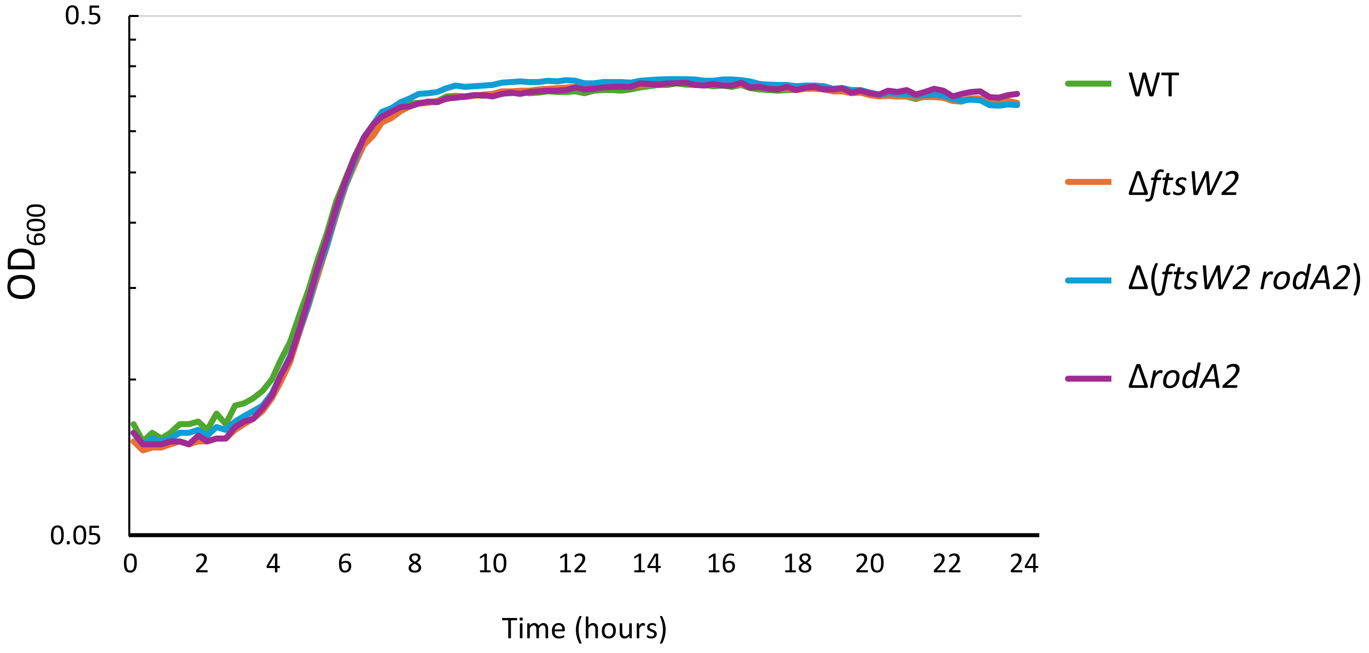

Supplemental Figure 2. Deletion of *ftsW2* or *rodA2* individually or together has no effect on growth. Growth of *E. faecalis* mutant strains lacking *ftsW2*, *rodA2*, or both compared to wild type (WT). The optical density at 600 nm (OD_{600}) of normalized cultures was determined every 15 minutes for 24 hours using a Bioscreen C plate reader. Data are representative of three biological replicates. Strains were WT = OG1; Δ*ftsW2* = MN31; Δ(*ftsW2 rodA2*) = JL529; ΔrodA2 = MN32.

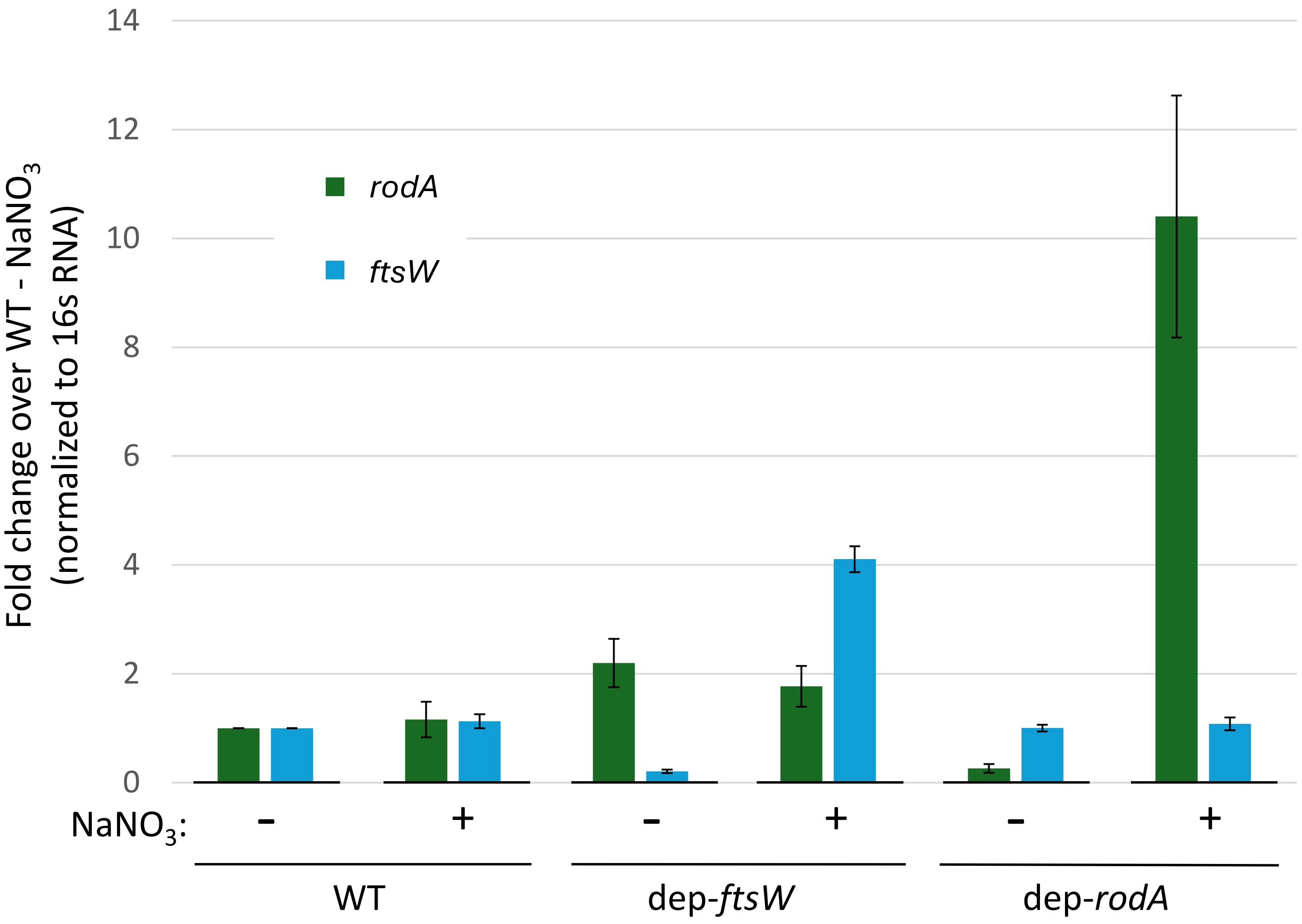

Supplemental Figure 3. *rodA* and *ftsW* transcript levels in the RodA or FtsW depletion strains. *ftsW* or *rodA* transcript levels from exponentially growing *E. faecalis* cells in the presence or absence of 25 mM $NaNO_3$, the inducer for RodA or FtsW expression, determined by quantitative reverse transcription PCR (RT-qPCR). Data represent the mean ± standard deviation of three biological replicates analyzed in technical triplicate. Strains were WT = OG1 (pJLL286); dep-*rodA* = MN34 (pMEN74); dep-*ftsW* = ML2 (pMEL34).

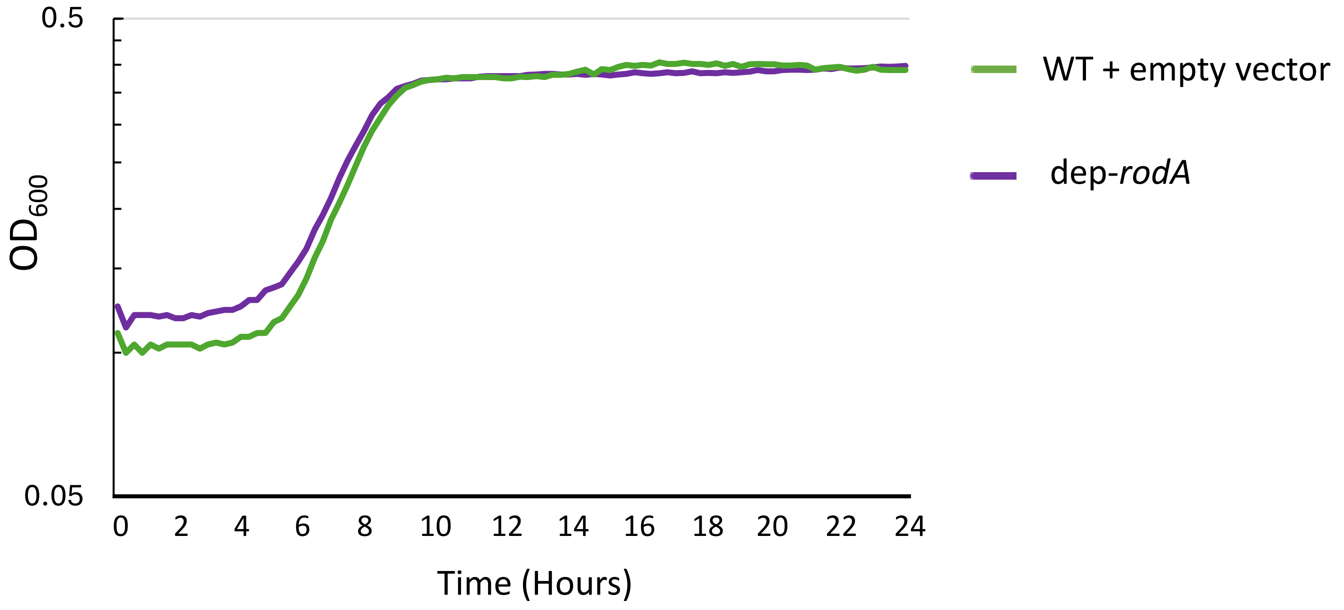

Supplemental Figure 4. The RodA depletion strain grows similar to WT in the absence of RodA inducer. Growth of the wild type (WT) and RodA depletion strain in the absence of $NaNO_3$, the inducer for RodA expression. The optical density at 600 nm (OD_{600}) of normalized cultures was determined every 15 minutes for 24 hours using a Bioscreen C plate reader. Data are representative of three biological replicates. "dep-*rodA*" denotes a RodA depletion strain (chromosomal deletion of *rodA* in the presence of a nitrate-inducible plasmid for RodA expression). Strains were WT + empty = OG1 (pJLL286); dep-*rodA* = MN34 (pMEN74).

Supplemental Table 1. FtsW2 and RodA2 are not required for resistance to various cell wall stressors.

Strain + Plasmid [b]	MIC_{Van} (µg/mL)[a]	MIC_{SDS} (% w/v)[a]	MIC_{Lys} (mg/mL)[a]	MIC_{Chloro} (% w/v)[a]	MIC_{Bac} (µg/mL)[a]	MIC_{Amp} (µg/mL)[a]	$MIC_{Cefepime}$ (µg/mL)[a]
WT	2	0.025	64	0.000488	64	0.5	32
Δ(*ftsW2 rodA2*)	2	0.025	64	0.000488	64	0.5	32
Δ*ftsW2*	2	0.025	n.d.[c]	n.d.	n.d.	n.d.	n.d.
Δ*rodA2*	2	0.025	n.d.	n.d.	n.d.	n.d.	n.d.

[a]The median minimal inhibitory concentration (MIC) of vancomycin (van), sodium dodecyl sulfate (SDS, percentage weight/volume), lysozyme (lys), chlorohexidine (chloro, percentage weight/volume), bacitracin (bac), ampicillin (amp), or cefepime determined from at least 2 biological replicates.

[b]Strains were WT (wild type) = OG1; Δ(*ftsW2 rodA2*) = JL529; Δ*ftsW2* = MN31; Δ*rodA2* = MN32.

[C] n.d. denotes not determined.

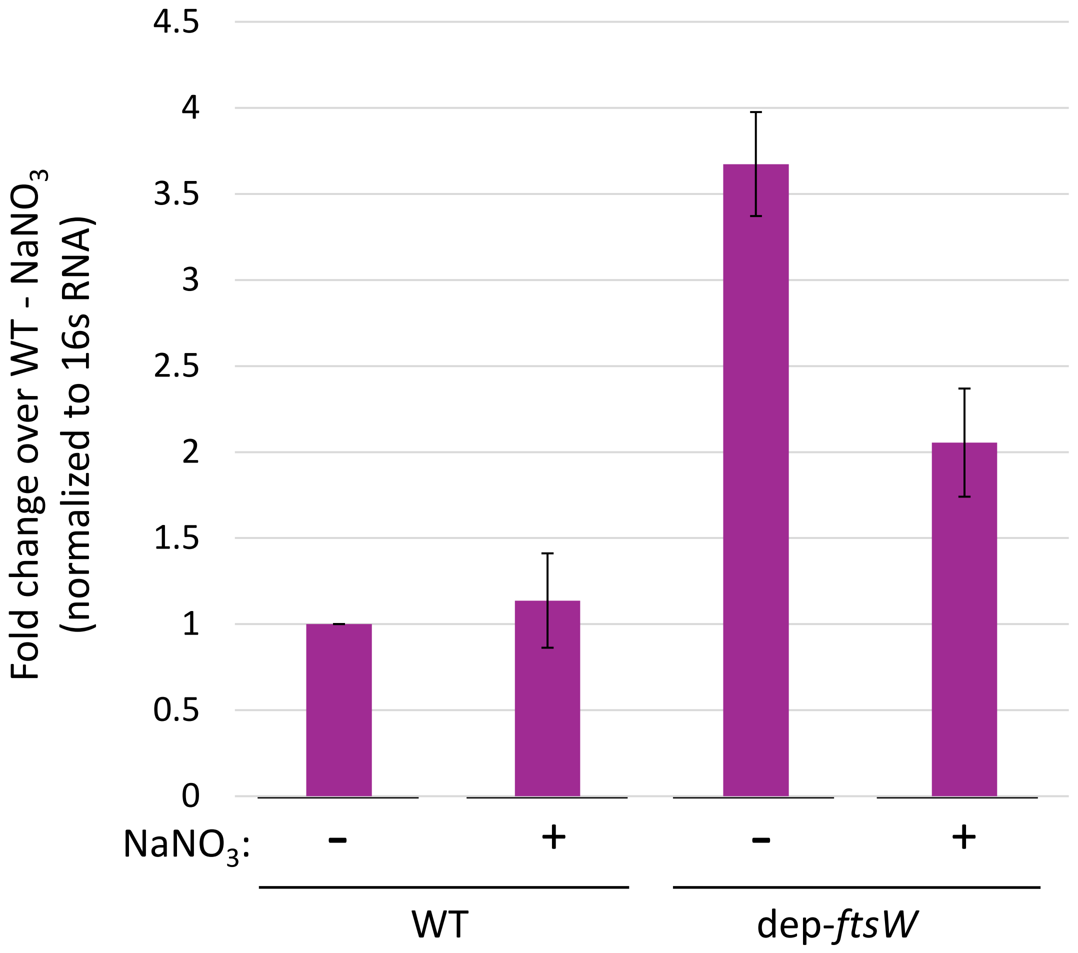

Supplemental Figure 5. Depletion of FtsW results in an increase in *croR* transcript. *croR* transcript levels from exponentially growing *E. faecalis* cells in the presence or absence of 25 mM $NaNO_3$ inducer determined by RT-qPCR. Data represent the mean ± standard deviation of three biological replicates analyzed in technical triplicate. Strains were WT = OG1 (pJLL286); dep-*ftsW* = ML2 (pMEL34).

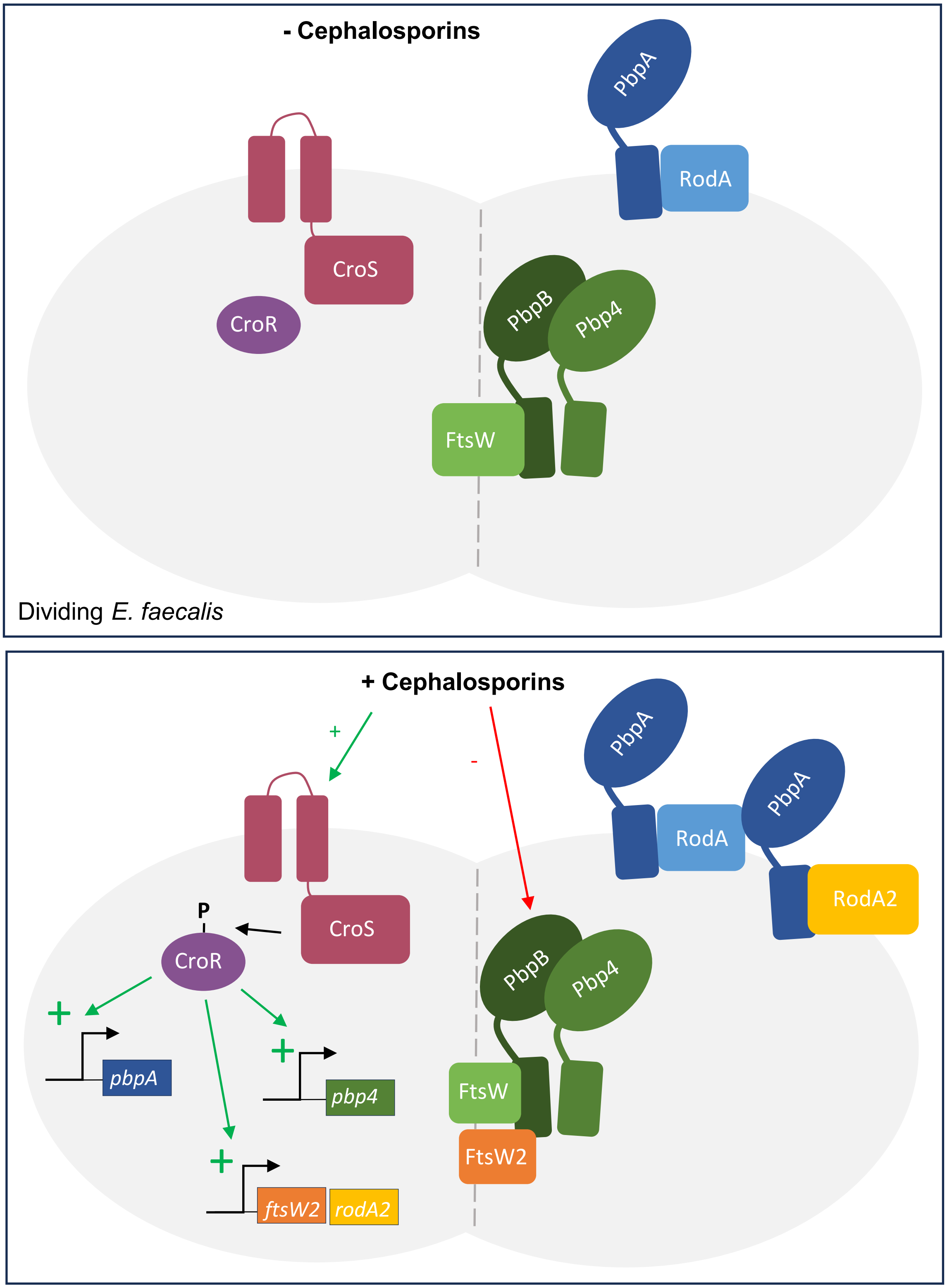

Supplemental Figure 6. Schematic depicting CroS/R-mediated upregulation of FtsW2/RodA2 to enhance cephalosporin resistance.

Supplemental Table 2. Bacterial strains and plasmids used in in this work.

Strain	Genotype or description	Source or reference
E. coli		
Top10	Routine cloning host	Lab stock
DH5α	Routine cloning host	Lab stock
Nico21	*E. coli* protein expression host	NEB
E. faecalis		
OG1	Wild-type reference strain	(1)
MN31	OG1 Δ*ftsW2* (Δ3-334)	This work
MN32	OG1 Δ*rodA2* (Δ15-318)	This work
JL529	OG1 Δ(*ftsW2 rodA2*)	This work
MN34	OG1 Δ*rodA* (Δ7-390) (pMEN74)	This work
ML2	OG1 Δ*ftsW* (pMEL34) (Δ3-401)	(2)
MN37	OG1 Δ(*ftsW2 rodA2*) Δ*ftsW* (pMEL34) (*ftsW* Δ3-401)	This work
MN38	OG1 Δ*croR* Δ*ftsW* (pMEL34) (*ftsW* Δ3-401)	This work
SB23	OG1 Δ*croR*	(3)
**Plasmids**		
pJH086	*E. faecalis* allelic-exchange vector (Cm^r, *repA V71G, lacZ pheS**)	(4)
pMEN75	Δ*ftsW2* (*E. faecalis*) deletion allele in pJH086	This work
pMEN73	Δ*rodA2* (*E. faecalis*) deletion allele in pJH086	This work
pJH087	Δ(*ftsW2 rodA2)* (*E. faecalis*) deletion allele in pJH086	This work
pJLL230	Δ*rodA* (*E. faecalis*) deletion allele in pJH086	This work
pMEL35	Δ*ftsW* (*E. faecalis*) deletion allele in pJH086	This work
pJLL286	Nitrate-inducible expression vector (Em^R)	(5)
pJRG9	Enterococcal expression vector carrying constitutive P23s promoter (Cm^R)	(3)
pET28a-his-smt3	*E. coli* protein expression vector	Volkman Lab, MCW
pET28b	*E. coli* protein expression vector	Novagen
pMEL32	pJRG9::*ftsW2-his*$_6$	This work
pMEL33	pJRG9::*rodA2-his*$_6$	This work
pMEL34	pJLL286::*ftsW-his*$_6$	(2)
pMEN74	pJLL286::*rodA*	This work
pMEL40	pET28a::*his*$_6$*-smt-pbp4*	(2)
pMEL42	pET28a::*his*$_6$*-smt-pbpB*	(2)
pMEL38	pET28a::*his*$_6$*-smt-pbpA*	(2)
pLMM25	pET28b-*his*$_6$*-pbpX* T36-P429	(2, 6)
pMEN86	pJRG9::*rodA2 D281A-his*$_6$	This work

References

1. Gold OG, Jordan HV, van Houte J. 1975. The prevalence of enterococci in the human mouth and their pathogenicity in animal models. Arch Oral Biol 20:473-IN15.

2. Nelson ME, Little JL, Kristich CJ. 2024. Pbp4 provides transpeptidase activity to the FtsW-PbpB peptidoglycan synthase to drive cephalosporin resistance in Enterococcus faecalis. Antimicrob Agents Chemother 68:e00555-24.

3. Snyder H, Kellogg SL, Skarda LM, Little JL, Kristich CJ. 2014. Nutritional Control of Antibiotic Resistance via an Interface between the Phosphotransferase System and a Two-Component Signaling System. Antimicrob Agents Chemother 58:957–965.

4. Kellogg SL, Little JL, Hoff JS, Kristich CJ. 2017. Requirement of the CroRS Two-Component System for Resistance to Cell Wall-Targeting Antimicrobials in Enterococcus faecium. Antimicrob Agents Chemother 61.

5. Mascari CA, Djoric D, Little JL, Kristich CJ. 2022. Use of an Interspecies Chimeric Receptor for Inducible Gene Expression Reveals that Metabolic Flux through the Peptidoglycan Biosynthesis Pathway is an Important Driver of Cephalosporin Resistance in Enterococcus faecalis. J Bacteriol 204:e00602-21.

6. Welsh MA, Taguchi A, Schaefer K, Van Tyne D, Lebreton F, Gilmore MS, Kahne D, Walker S. 2017. Identification of a Functionally Unique Family of Penicillin-Binding Proteins. J Am Chem Soc 139:17727–17730.
